# miR-182/183-Rasa1 axis induced macrophage polarization and redox regulation promotes repair after ischemic cardiac injury

**DOI:** 10.1016/j.redox.2023.102909

**Published:** 2023-09-29

**Authors:** Yijun Yang, Jaslyn Johnson, Constantine D. Troupes, Eric A. Feldsott, Lindsay Kraus, Emily Megill, Zilin Bian, Ngefor Asangwe, Tabito Kino, Deborah M. Eaton, Tao Wang, Marcus Wagner, Lena Ma, Christopher Bryan, Markus Wallner, Hajime Kubo, Remus M. Berretta, Mohsin Khan, Hong Wang, Raj Kishore, Steven R. Houser, Sadia Mohsin

**Affiliations:** a Cardiovascular Research Center (CVRC), Temple University Lewis Katz School of Medicine, PA, United States; b Center for Metabolic Disease Research (CMDR), Temple University Lewis Katz School of Medicine, PA, United States; c Center for Translational Medicine, Temple University Lewis Katz School of Medicine, PA, United States; d Tandon School of Engineering, New York University, NY, United States; e Division of Cardiology, Medical University of Graz, 8036, Graz, Austria

**Keywords:** Cortical bone stem cells, Extracellular vesicles, Cardiac repair, Immune modulation

## Abstract

Few therapies have produced significant improvement in cardiac structure and function after ischemic cardiac injury (ICI). Our possible explanation is activation of local inflammatory responses negatively impact the cardiac repair process following ischemic injury. Factors that can alter immune response, including significantly altered cytokine levels in plasma and polarization of macrophages and T cells towards a pro-reparative phenotype in the myocardium post-MI is a valid strategy for reducing infarct size and damage after myocardial injury.

Our previous studies showed that cortical bone stem cells (CBSCs) possess reparative effects after ICI. In our current study, we have identified that the beneficial effects of CBSCs appear to be mediated by miRNA in their extracellular vesicles (CBSC-EV). Our studies showed that CBSC-EV treated animals demonstrated reduced scar size, attenuated structural remodeling, and improved cardiac function versus saline treated animals. These effects were linked to the alteration of immune response, with significantly altered cytokine levels in plasma, and polarization of macrophages and T cells towards a pro-reparative phenotype in the myocardium post-MI. Our detailed *in vitro* studies demonstrated that CBSC-EV are enriched in miR-182/183 that mediates the pro-reparative polarization and metabolic reprogramming in macrophages, including enhanced OXPHOS rate and reduced ROS, via Ras p21 protein activator 1 (RASA1) axis under Lipopolysaccharides (LPS) stimulation. In summary, CBSC-EV deliver unique molecular cargoes, such as enriched miR-182/183, that modulate the immune response after ICI by regulating macrophage polarization and metabolic reprogramming to enhance repair.

## Non-standard abbreviations and acronyms:

CBSCs: Cortical Bone Derived Stem Cells
CBSC-EV: CBSCs derived extracellular vesicles
ICI: Ischemic Cardiac Injury
MI: Myocardial Infarction
CXCL: The chemokine (C-X-C motif) ligand
MCSF: Macrophage colony stimulating factor
CCL: C–C motif chemokine ligand
LPS: Lipopolysaccharide
IL-6: Interleukin 6
IL-10: Interleukin 10
IL-18: Interleukin 18
NS: No significance
LVID: Left ventricular internal diameter
LVEDV: Left ventricular end-diastolic volume
LVESV: Left ventricular end-systolic volume
LV mass: Left ventricular mass
BMDMФs: Bone marrow derived macrophages
PCA: Principal component analysis
ROS: Reactive oxygen species
Rasa1: Ras p21 protein activator 1
c-Myc: Avian myelocytomatosis virus oncogene cellular homolog
SOD2: mitochondrial superoxide dismutase

## 1 Introduction

Ischemic heart disease is one of the most common causes of mortality and its prevalence continues to rise in the United States [1]. Current pharmacological treatment is designed to be ‘damage limiting’, yet unable to effectively prevent adverse remodeling and scar formation after ICI. Cell-based therapies for cardiac repair and regeneration have been tested as an alternative to existing pharmacological and surgical interventions [2,3]. Adoptive transfer of progenitor cells into an injured myocardium have been shown to lead modest improvement in cardiac pump function, however, the mechanisms of cell therapy remain unclear, and the limitations of cell therapy exist such as low retention rate after transplantation [[4], [5], [6]]. Recent studies suggest that factors secreted by transplanted cells, such as extracellular vesicles, can modulate molecular signaling complexes in target cells to improve wound healing and reduce scar formation [7]. These findings suggest that paracrine factors released by donor cells may possess equivalent reparative effects as their parent cells. Nevertheless, growing consensus in the field suggests that most if not all of the donated stem cells are lost soon after their introduction [8,9], yet their beneficial effects persist, thereby meriting development of new strategies that harness the power of cell paracrine factors.

Cardiac wound healing after ICI is a dynamic process comprising of precise activation of various immune cells to remove damaged tissue and replace it with scar tissue. Unlike other tissues, cardiac scar persists and can expand resulting in adverse cardiac remodeling. ICI kills cardiac myocytes and supportive tissue [10,11], inducing release of a constellation of pro-inflammatory cytokines and chemokines from the dead tissue that triggers an inflammatory response and subsequent recruitment and activation of cardiac immune cells to the site of injury [12]. Cell therapy added to the post-MI heart via adoptive transfer are often rapidly lost [9,[13], [14], [15]], and these donated cells are also exposed to pro-inflammatory cytokines, chemokines and immune cells that accumulated in the injured myocardium. Whether this early loss of transplanted cells is connected to the cardiac inflammatory response profile prevalent in the heart post-MI remains poorly understood. Ideally, donated cells that can withstand the onslaught of detrimental factors and at the same time possess the ability to manipulate inflammatory responses to transform the cardiac environment into a pro-reparative state would represent a viable choice for cell-based therapies.

Recently, we have identified CBSCs as a novel stem cell population from the bone stroma, possessing cardiac repair abilities as shown in small and large animal models of MI [16,17]. Previous studies showed that CBSCs injected into the MI border zone were able to restrict infarct size and improve cardiac pump structure and function, which is superior to other donated cells [16,17]. CBSCs treatment in a swine model of MI led to an alteration of immune cell patterns towards a more pro-reparative state in the myocardium [18]. Moreover, CBSCs secrete several unique growth factors and paracrine effectors with potential cardioprotective properties [17]. Among the secreted cell factors, extracellular vesicles (EVs) have acquired prominence due to their enriched bioactive cargos including micro-ribonucleic acids and proteins. Our previous study suggested that CBSC-EV recapitulate the effects of CBSCs treatment at 24 h after ICI [19]. The hypothesis that CBSC-EV can recapitulate the reparative effect of CBSCs in a long-term model and modulate cardiac immune response to improve post-MI wound healing has not been tested and is the focus of this research.

## 2 Results

### 2.1 Characterization and functional validation of CBSC-EV

CBSC-EV were isolated from parent CBSCs (Supplementary Fig. 1A) that were cultured in media containing FBS depleted of extracellular vesicles (System Bio, Exo-FBS-250A-1) for 3 days. CBSC-EV samples have a typical EV size distribution of <120 nm using Nanosight, and EV preparations contained ∼7-8 x10^8^ particles/ml (Supplementary Fig. 1B). Electron micrographs also validated CBSC-EV that showed typical morphology and size (Supplementary Fig. 1C). Additionally, CBSC-EV contained a typical expression of proteins including CD9 and CD63 (Supplementary Fig. 1D). Collectively, these data showed that the techniques we employed isolated EVs from CBSCs with a normal size distribution of 50–150 nm as reported previously [20].

To investigate the reparative capacity of CBSC-EV related to cardiomyocyte protection and angiogenesis, we performed a series of *in-vitro* experiments. We first challenged neonatal rat ventricular myocytes (NRVMs) with oxidative stress. Treatment of NRVMs with 50 μm H_2_O_2_ for 4 h induced myocyte apoptosis which was rescued by CBSC-EV treatment. CBSC-EV significantly reduced the number of TUNEL positive NRVMs by 2-fold versus control (Supplementary Fig. 1E). Moreover, we plated HUVECs on matrigel and found that CBSC-EV treatment led to a 2.2-fold increase in tube formation compared with controls (Supplementary Fig. 1F). Together these data suggest CBSC-EV possess the capacity to protect cardiomyocytes from apoptosis and promote neovascular angiogenesis *in-vitro*.

### 2.2 CBSCs and CBSC-EV equivalently improve cardiac function after injury

**Fig. 1:**
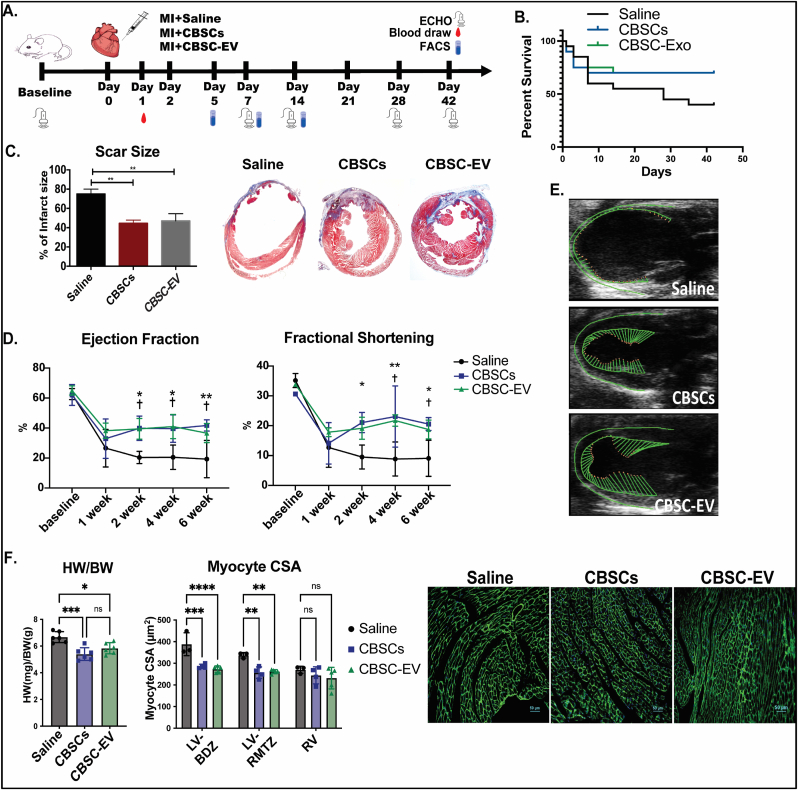
Functional assessment after CBSCs and CBSC-EV transplantation post-MI. A. Schema for experimental design; Mice received Saline, CBSCs or CBSC-EV treatment right after myocardial infarction surgery. Serial echocardiography measurements were performed at baseline, 1 week, 2 weeks, 4 weeks and 6 weeks post-MI. B. Kaplan-Meier curves of survival rate post-MI. C. Reduction in infarct size in CBSCs and CBSC-EV treated animals determined by Masson's Trichrome staining in heart tissues, **p < 0.01, ***p < 0.001. D. Ejection Fraction and Fractional Shortening was improved in mice treated with CBSCs and CBSC-EV compared to saline 2–6 weeks post-MI. (n = 6 animals per group), *p < 0.05, **p < 0.01, CBSCs versus Saline, †*p* < 0.01 CBSCs-EV versus Saline. E. Representative images of vector diagrams by speckle tracking analysis were shown. F. Heart weight to body weight ratio at terminal. With cardiomyocyte cross-sectional area (CSA) determined with wheat germ agglutinin (WGA) staining in left ventricle boarder zone (LV-BDZ), left ventricle remote zone (LV-RMTZ) and right ventricle (RV) respectively. *p < 0.05, **p < 0.01, ***p < 0.001, ****p < 0.0001.

Permanent LAD occlusion induced myocardial infarction (MI) model was performed in mice to assess whether CBSC-EV could recapitulate the reparative effects of CBSCs and improve wound healing after MI. CBSCs (4000 cells/μL), CBSC-EV (120 μg protein with 7X10^8^ particles/ml) or saline were directly injected into the border zone at the time of MI as described previously [16]. The schema for the MI experiment is delineated in Fig. 1A. Mice that received CBSCs or CBSC-EV showed 1.75-fold increase in survival rate 42-days post-MI (Fig. 1B) and a significant reduction by 47% in scar size determined by Masson's Trichrome staining (Fig. 1C). CBSCs and CBSC-EV transplantation also led to significant improvement in cardiac function determined by echocardiography (ECHO). ECHO derived parameters including ejection fraction and fractional shortening were significantly improved versus saline treated animals starting from 2 weeks after MI (p < 0.05 CBSCs and CBSC-EV versus Saline; non-significant for CBSCs versus CBSC-EV) (Fig. 1D). Moreover, CBSCs and CBSC-EV treatments led to attenuated cardiac hypertrophy and chamber dilation, with significantly reduced left ventricular (LV) mass, end-diastolic LV volume (LVEDV) and end-systolic LV volume (LVESV) 6 weeks post-MI (Supplementary Fig. 2A). Vector diagrams by speckle tracking analysis (Fig. 1E), 2D M-mode images and three-dimensional regional wall strain diagrams (Supplementary Fig. 2B) illustrate improved contractility, better myocardium synchrony and attenuated remodeling in CBSCs and CBSC-EV treated animals 6 weeks post-MI. Heart weight to body weight ratios were significantly smaller in CBSCs and CBSC-EV treated animals versus saline, documenting attenuated post-MI hypertrophy at organ level (Fig. 1F). At the cellular level, myocyte cross-sectional area (CSA) (μm [2]) was significantly smaller in CBSCs and CBSC-EV versus saline treated hearts in both the LV border zone and LV remote zone, although with no difference was found in the right ventricle (Fig. 1F). Overall, our *in-vivo* study suggests that CBSC-EV produce the same salutary effects as CBSCs in restricting infarct expansion, preserving cardiac function and attenuating deleterious structural remodeling after ICI.

### 2.3 CBSCs and CBSC-EV restrict cell death shortly after ICI

**Fig. 2:**
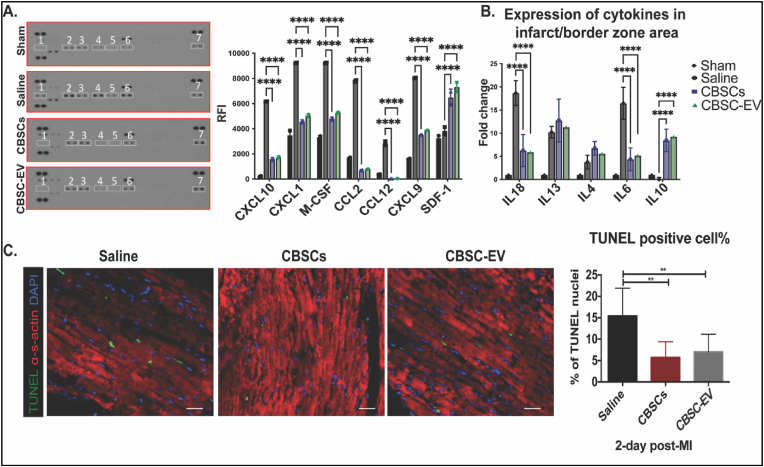
CBSCs and CBSC-EV modulated cytokine profile and restricted cell deaths acutely post-MI. A. Plasma analysis of cytokine protein levels 24 h after MI; 1) CXCL10, 2) CXCL1, 3) M-CSF, 4) CCL2, 5) CCL12, 6) CXCL9, 7) SDF-1, ****p < 0.0001. B. Gene expression levels of pro-inflammatory cytokines such as IL-18, IL-6, and anti-inflammatory cytokines such as IL-10 in infarct border zone area 24 h after MI (n = 5 animals in Saline, CBSCs, CBSC-EV treated groups), ****p < 0.0001. C. Myocardium apoptotic cells 2 days after MI measured by TUNEL staining (TUNEL: green; DAPI: blue; α-sarcomeric actin: red). **P < 0.01 versus saline. (For interpretation of the references to colour in this figure legend, the reader is referred to the Web version of this article.)

Acutely after MI, the injured myocardium releases pro-inflammatory cytokines which contribute to the death of resident cardiomyocytes [21]. In the present study, we investigated cytokine levels 24 h post-MI in the circulation and injured site. Plasma analysis of cytokines showed that CBSCs and CBSC-EV treated animals 24 h post-MI had significantly lower levels of pro-inflammatory chemokines including CXCL10, CXCL1, M-CSF, CCL2, CCL12, and CXCL9 compared to saline. In parallel, expression of SDF-1 was elevated in CBSCs and CBSC-EV treated animals (Fig. 2A). Gene expression levels of pro- and anti-inflammatory cytokines in heart tissues from the infarct/border area 24 h post-MI were also analyzed, which showed significantly reduced expression of pro-inflammatory factors such as IL18 and IL6, and increased expression of an anti-inflammatory factor (IL10) in CBSCs and CBSC-EV treated mice (Fig. 2B). Additionally, CBSCs and CBSC-EV treated hearts had 2.5-fold less TUNEL + cells two days post-MI (Fig. 2C), consistent with the cardioprotective effects observed *in-vitro*. These data suggest that CBSC-EV recapitulate the reparative effects of CBSCs acutely after ICI by regulating systemic and local cytokine levels augmenting cardiac reparative processes.

### 2.4 CBSCs and CBSC-EV promote pro-reparative immune response post-MI

**Fig. 3:**
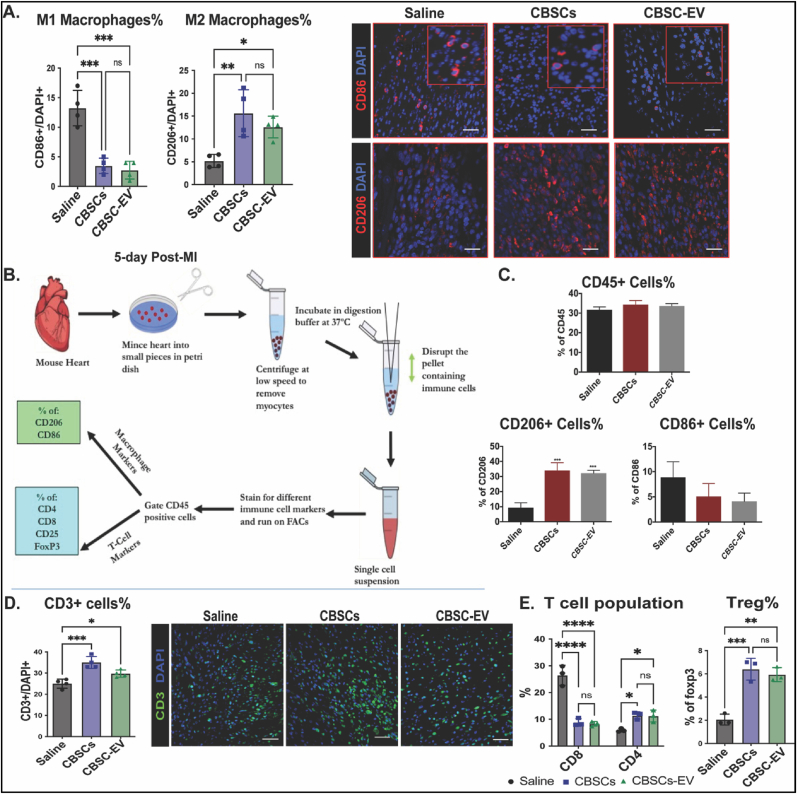
CBSCs and CBSC-EV promote pro-reparative immune responses in the myocardium post-MI. A. Percentage of CD86 (M1 macrophage marker) positive cells and CD206 (M2 macrophage marker) positive cells among total DAPI determined by immune fluorescent staining of left ventricular heart tissues 5-day post-MI, *p < 0.05，**p < 0.01, ***p < 0.001. B. Schema of cardiac immune cell isolation and Flow cytometry quantification performed 5, 7 or 14-day post-MI. C. Percentage of CD45, CD206 and CD86 positive cells respectively determined by Flow cytometry, ***p < 0.001 (n = 3 animals per group). D. CBSCs and CBSC-EV treatment increased CD3 positive cells infiltration after MI measured by immunofluorescence analysis. E. Percentage of CD8, CD4 in total CD45 positive cell population and FoxP3 positive cells in CD4 positive cell population 14 days post-MI determined by Flow cytometry. *P < 0.05, **P < 0.01, ***P < 0.001, ****P < 0.0001 versus saline.

Previous studies suggest around 4–14 days after MI, the injured myocardium undergoes a reparative phase, during which immune cells including both myeloid and lymphoid populations play a crucial role in removing damaged cardiac tissue and replacing this tissue with scar [22,23]. Thus, the interaction of transplanted CBSCs and CBSC-EV with the immune cells is the major focus of this study. Since the initial expansion of monocytes peaks around day 5 after MI [24], we analyzed the monocyte population in our study at 5 days post-MI. Histological analysis showed a 3.7-fold decrease in the percentage of CD86 (a marker for pro-inflammatory M1 macrophages) positive cells and an increase in the percentage of CD206 (a marker for anti-inflammatory M2 macrophages) positive cells in the infarct/border zone with CBSCs and CBSC-EV treatment compared with saline 5 days post-MI (Fig. 3A). To validate this finding, cardiac immune cells were isolated from the whole heart and analyzed by Flow cytometry. The strategy for isolation of cardiac immune cells is delineated in Fig. 3B (Gating strategy for Flow cytometry is listed in Supplementary Fig. 3). Expression of the pan-hematopoietic marker CD45 remained unchanged between CBSCs and CBSC-EV versus saline treated animals 5 days post-MI (Fig. 3C). Consistent with histology, the percentages of CD206 positive cells were significantly increased in both CBSCs and CBSC-EV treated animals, accompanied with a reduction of CD86 positive cells at 5 days post-MI as determined by Flow cytometry analysis (Fig. 3C). The infiltration of lymphoid cells is considered to peak at day 7 post-MI [25]. At 7-day post-MI, the percentages of CD8 (cytotoxic T cell marker) positive cells were 2-fold decreased significantly in animals treated with CBSCs and CBSC-EV, with a trend of increased CD4 (T-helper cells) expressing cells compared with saline determined by Flow cytometry analysis (Supplementary Fig. 4). We observed a more prominent change of T cell population at day 14 post-MI. Immunostaining of CD3 revealed slightly increased T cell infiltration in CBSCs and CBSC-EV treated mice 14-days post-MI (Fig. 3D). Flow cytometry analysis determined that the percentages of CD8^+^ T cells were 68% lower, along with a 2-fold significant increase in the number of CD4^+^ T cells subset in hearts from CBSCs and CBSC-EV transplanted animals versus Saline (Fig. 3E). Moreover, there was a 3-fold increase in foxp3+ T-helper cells in CBSCs and CBSC-EV transplanted hearts, indicating an increased Treg population (Fig. 3E). Collectively, these data suggest that CBSCs and CBSC-EV modulate the cardiac immune cells response in the heart after myocardial injury, including macrophages and T cells, and lead to a transition towards an anti-inflammatory, pro-reparative immune cell subsets. Moreover, immunofluorescent staining of heart sections from CBSCs or CBSC-EV treated mice had increased numbers of cells that were positive for SM22 and von Willebrand factor (vWF) 6 weeks post-MI, indicating enhanced angiogenesis (Supplementary Fig. 5A). However, there were no differences in the percentage of EdU positive cells in the infarct border zone among groups (Supplementary Fig. 5B).

### 2.5 CBSC-EV modulates macrophage phenotype *in-vitro* through its unique transcriptome cargo

**Fig. 4:**
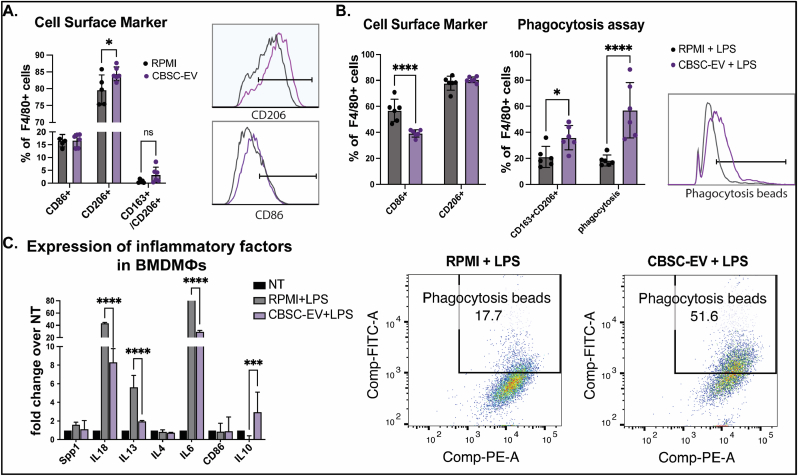
CBSCs derived extracellular vesicles (CBSC-EV) regulates bone marrow derived macrophages (BMDMs) polarization and phagocytosis *in vitro*. A. CBSC-EV treated bone marrow derived macrophages (BMDMФs) showed increased CD206+ population compared to control RPMI media without LPS stimulation determined by Flow cytometry, *p < 0.05. B. Under LPS stimulation, CBSC-EV treated BMDMs showed decreased CD86^+^ macrophage population, increased CD163/CD206 double positive macrophages and enhanced phagocytosis capacity determined by Flow cytometry, *p < 0.05, ****p < 0.0001. C. Expression levels of pro- and anti-inflammatory genes determined by qRT-PCR with or without LPS stimulation in BMDMs, ***p < 0.001, ****p < 0.0001.

The ability of CBSCs to modulate immune cell phenotypes was further validated *in-vitro* by culturing macrophages isolated from the bone marrow (BMDMФs) cultured with CBSC-EV or control RPMI media. Without inflammatory stimulation, CBSC-EV induced slightly increased percentage of CD206+ M2 macrophages determined by Flow cytometry analysis (Fig. 4A, Gating strategy for Flow cytometry is listed in Supplementary Fig. 6). When we challenged the cultured BMDMs with 18 h treatment of 50 ng/ml Lipopolysaccharides (LPS), CBSC-EV significantly reduced the percentage of CD86^+^ M1 macrophages and induced a polarization towards a CD163/CD206 double positive phenotype (Fig. 4B). BMDMs cultured with CBSC-EV also showed a 3-fold increase in uptake of FITC conjugated phagocytosis beads compared with RPMI (Fig. 4B). Gene expression level of pro- and anti-inflammatory cytokines in BMDMs were determined by qRT-PCR. CBSC-EV significantly reduced the expression of pro-inflammatory cytokines such as IL-18 (80% decrease) and IL-6 (by 7.7-fold), and increased the expression level of anti-inflammatory IL-10 (by 138-fold) compared with BMDMs cultured in control media (Fig. 4C). These findings suggest CBSC-EV promote macrophages polarization towards a reparative phenotype with enhanced phagocytosis efficacy *in-vitro*.

**Fig. 5:**
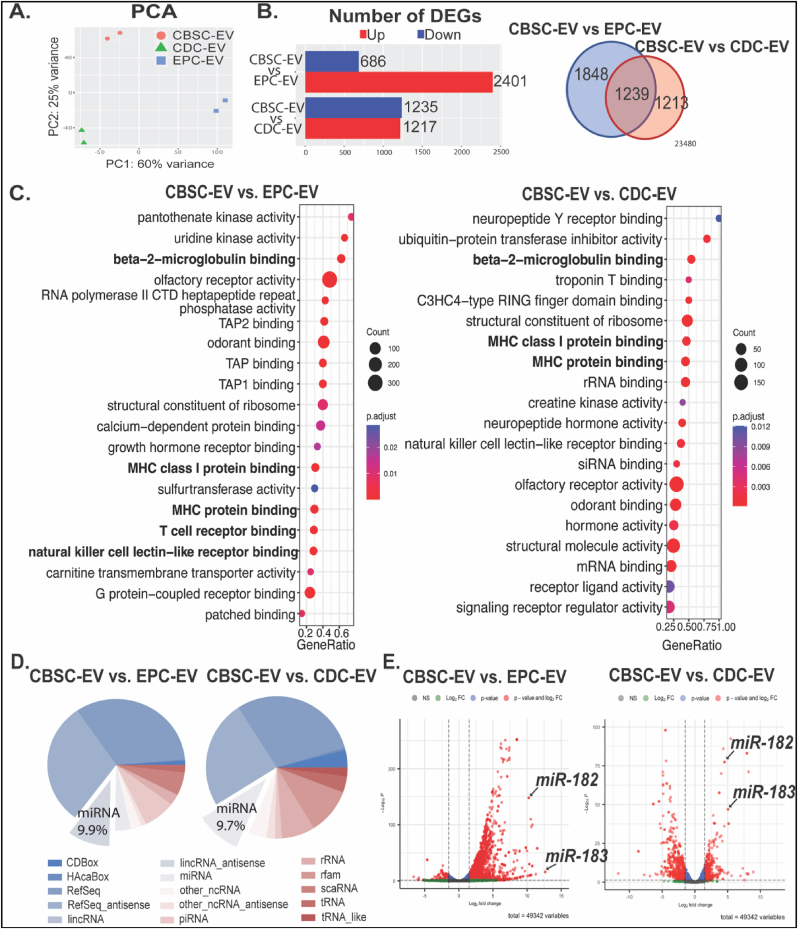
CBSCs derived extracellular vesicles (CBSC-EV) possess significantly different transcriptome profile compared with extracellular vesicles isolated from endothelial progenitor cells (EPCs) and cardiosphere-derived cells (CDCs). A. Principal component analysis (PCA) of the transcriptome profiles of CBSC-EV, CDC-EV and EPC-EV. B. Number of differentially expressed genes (DEGs) when comparing CBSC-EV versus EPC-EV and CDC-EV respectively. Venn diagram depicted the number of overlapped DEGs between comparisons. C. Top GO derived from gene ontology enrichment analysis of molecular function comparing CBSC-EV versus EPC-EV and CDC-EV respectively. D. Pie-charts depicted the compositions of DEGs, miRNA counts 9.9% and 9.7% of DEGs respectively. E. Volcano plots representing relative gene expression levels with fold change (x-axis) and p value (y-axis) comparing CBSC-EV versus EPC-EV or CDC-EV. MiR-182 and miR183 rank top of the most significant upregulated genes.

Our previous study suggested that CBSCs had enhanced reparative capacity when compared to other progenitor cells such as cardiosphere-derived cells (CDCs) and mesenchymal stem cells [16,17]. To investigate the underlying mechanisms of CBSC-EV mediated immunomodulation effects and compare the difference in transcriptome cargo between EVs isolated from different progenitor cells, we performed RNA sequencing analysis of extracellular vesicles derived from CBSCs, endothelial progenitor cells (EPCs) and CDCs respectively. We determined that the transcriptome of CBSC-EV is largely distinct from EPC-EV and CDC-EV represented by PCA analysis (Fig. 5A) and heatmaps of the top 480 most distinct genes (Supplementary Fig. 7A). CBSC-EV had 3087 and 2452 differentially expressed genes (DEGs) when compared to EPC-EV and CDC-EV respectively (Fig. 5B). Venn diagram suggested there were 1239 DEGs overlapped between comparisons (Fig. 5B). Gene ontology enrichment analysis on molecular function of DEGs suggested significant enrichment in immune cell receptor binding related GO terms, such as beta-2-microglobulin binding, TAP binding and T cell receptor binding (Fig. 5C), suggesting that these DEGs were enriched in the regulation of immune cell surface receptor bindings, and may be involved in the intracellular signaling. Among the 2 comparisons determined DEGs, non-coding miRNAs take up about 9.9% and 9.7% percent respectively (Fig. 5D). More importantly, miRNAs rank at the top of most variable genes depicted by heatmap (Supplementary Fig. 6B). We identified miRNA-182 and miRNA-183 as two of the most significantly upregulated DEGs in CBSC-EV compared with both EPC-EV and CDC-EV (Fig. 5E, Supplementary Fig. 6B). Together these data suggest that CBSC-EV has a unique transcriptome cargo that are enriched in immune cell surface receptor bindings, which may underlie its immune modulation capacity.

### 2.6 miR-182/183 enriched in CBSC-EV mediate immune cells phenotype after injury

**Fig. 6:**
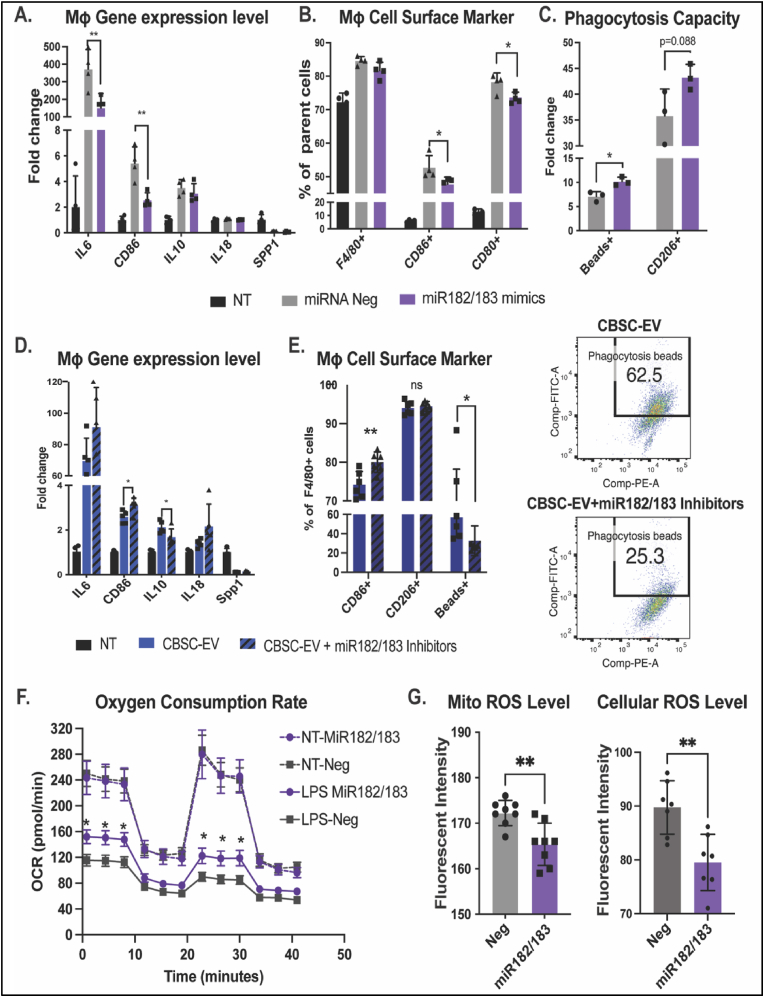
CBSCs derived extracellular vesicles (CBSC-EV) modulates macrophage polarization through miR-182/183. A. Gene expression levels of IL6, CD86, IL10, IL18 and Spp1 from bone marrow derived macrophages (BMDMs) in culture media (NT), or after treated with LPS with miRNA negative control or miR-182/183 mimics. B. Percentage of F4/80 positive cells, CD86 and CD80 positive cells in F4/80+ populations were determined with Flow cytometry analysis. C. Percentage of phagocytotic beads positive cells and CD206 positive cells in F4/80+ populations determined with Flow cytometry analysis. D. Gene expression levels of IL6, CD86, IL10, IL18 and Spp1 BMDMs in culture media (NT), or after treated with LPS and CBSC-EV with or without miR-182/183 inhibitors. E. Percentage of CD86, CD206 and phagocytosis beads (FITC) positive cells were determined with Flow cytometry analysis. F. Seahorse assay determined the OXPHOS rate in BMDMs treated without LPS or with 24 h LPS treatment, that have either miRNA negative control or miR-182/183 mimics treatment. G. Mitochondrial ROS and cellular ROS level from miRNA negative control and miR-182/183 mimics pre-treated BMDMs after 3 h LPS treatment. *p < 0.05, **p < 0.01, ***p < 0.001.

To further investigate the role of miR-182 and miR-183, which are significantly enriched in CBSC-EV compared with EPC-EV and CDC-EV, we challenged BMDMs with LPS together with miR-182/183 mimics or miRNA scramble negative control (Neg) for 18 h. BMDMs without LPS treatment (NT) was used as a control. Gene expression levels of IL6 and CD86 were significantly downregulated in miR-182/183 mimics treated cells with 2.5-fold and 2.1-fold respectively versus negative control (Fig. 6A). Flow cytometry analysis suggested a reduction of CD86^+^ and CD80^+^ population with miR-182/183 mimics treatment (Fig. 6B). When BMDMs were supplemented with phagocytosis beads, we observed enhanced phagocytosis and a trend of increased CD206+ populations with miR-182/183 treatment determined by Flow cytometry (Fig. 6C). Additionally, treatment of miR-182/183 inhibitors can block the immune modulation effect of CBSC-EV. BMDMs cultured with CBSC-EV or CBSC-EV supplemented with miR-182/183 inhibitors were challenged with LPS for 18 h. Inhibition of miR-182/183 in CBSC-EV induced 1.3-fold increase of IL-6, 1.2-fold increase of CD86 and 1.3-fold decrease in IL-10 gene expression levels determined by qRT-PCR (Fig. 6D). Flow cytometry analysis determined a significant increase of CD86^+^ cells and 1.7-fold decrease of phagocytic cells with miR-182/183 inhibitor treatment (Fig. 6E). Together these data suggest that miR-182/183 partially contribute to the immune modulation effect of CBSC-EV, if not all, in cultured BMDMs. Moreover, we observed that miR-182/183 treatment also promotes BMDMs metabolic reprograming to an anti-inflammatory phenotype. LPS stress attenuated oxygen consumption in BMDMs, while miR-182/183 partially preserved the oxygen consumption rate under LPS stress (Fig. 6F). Interestingly, miR-182/183 treatment attenuated both mitochondrial ROS production and cellular ROS production in miR-182/183 treated BMDMs after 3 h LPS treatment (Fig. 6G, Supplementary Fig. 8). Additionally, we also tested the effect of miR-182/183 on spleen derived T cells *in-vitro*. miR-182/183 mimics treatment reduced the percentage of CD8^+^ population and increased CD25+CD4^+^ population (Treg phenotype). Moreover, miR-182/183 mimics treatment led to a trend of increased FoxP3+ Treg (Supplementary Fig. 9). However, the differences are small between miR-182/183 mimics and negative control.

### 2.7 miR-182/183 regulate macrophages polarization through RASA1 axis

**Fig. 7:**
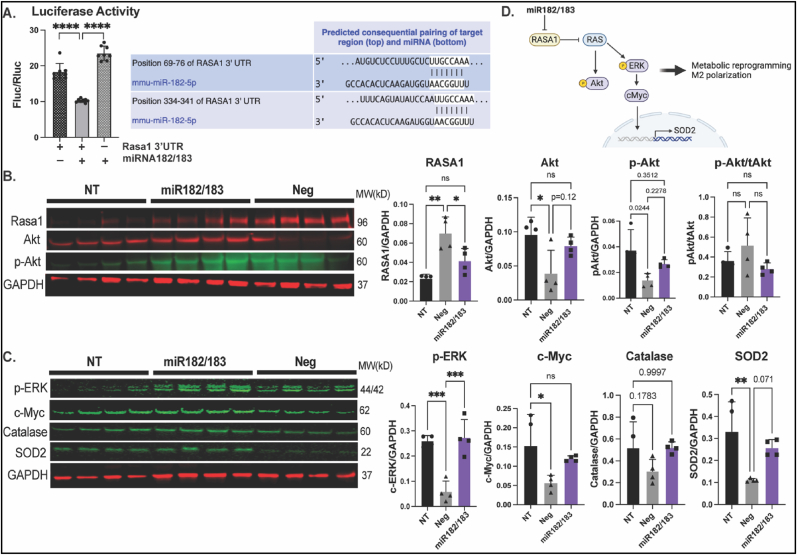
miR-182/183 inhibits Rasa1 and activates downstream pathways. A. Ras p21 activator protein 1 (Rasa1) is the predicted target gene of miR-182-5p. Inhibition of Rasa1 by miR-182/183 was confirmed by luciferase assay in HEK293 cells. B. Protein level of Rasa1, Akt and phosphorylated-Akt (p-Akt) in BMDMs after treated with LPS and miRNA negative control of miR-182/183 mimics were quantified. BMDMs without LPS treatment served as non-treatment (NT) control. C. Protein level of pERK, c-Myc, catalase and SOD2 in BMDMs after treated with LPS and miRNA negative control of miR-182/183 mimics were quantified. BMDMs without LPS treatment served as non-treatment (NT) control. D. Illustration of proposed signaling pathways of miR-182/183. *p < 0.05, **p < 0.01, ***p < 0.001.

Rasa1 is one of the predicted targets of miR-182. The inhibition of RASA1 by miR-182/183 treatment was confirmed by luciferase assay (Fig. 7A). We found that the protein level of RASA1 was 3-fold increase in LPS treated compared with non-treated (NT) BMDMs. miR-182/183 treatment largely attenuated the increase of RASA1 under LPS stress (Fig. 7B). Rasa1 inhibits downstream Akt and ERK activation through the inhibition of Ras [26]. We found that LPS stress induces decreased both Akt and phosphorylated Akt (p-Akt) levels. miR-182/183 treatment preserved Akt and p-Akt protein expression sunder LPS stress (Fig. 7B). LPS treatment also attenuated phosphorylated ERK (p-ERK) by 4.4-fold, miR-182/183 treatment significantly preserved the protein level of p-ERK (Fig. 7C). We then examined c-Myc, which is considered as a key regulator for M2 polarization that is downstream of ERK. We found LPS stress leads to 3-fold decrease in c-Myc protein level, and this can be salvaged by miR-182/183 treatment (Fig. 7C). To understand the reduction of ROS levels in miR-182/183 treated BMDMs, we tested protein levels of Catalase and SOD2 and observed preserved Catalase and SOD2 levels in miR-182/183 treated cells under LPS stress (Fig. 7C). Our data suggests that miR-182/183 treatment activates RAS downstream signaling through Rasa1 inhibition (Fig. 7D).

## 3 Discussion

### 3.1 CBSC-EV induced reparative effects equivalent to CBSCs after MI

Our previous studies showed that CBSCs significantly preserve cardiac function and reduce scar size after ischemic injury in both mouse and swine models [16,27]. Extracellular vesicles, which are enriched in proteins, mRNAs and non-coding RNAs, are considered as a vital secretome that mediate the effect of parental cells [7] and may possess the same salutary effects as their parental cells [28,29]. In this study, we first confirmed that CBSC-EV possess reparative effects that are equivalent to CBSCs after MI in a mouse model, by significantly improving survival, cardiac function, attenuating scar expansion and pathological remodeling to the same extent at 6 weeks post-MI (Fig. 1). Collectively, these data suggest that extracellular vesicles released by CBSCs recapitulate the salutary effects of their parental cells post-MI and thus may serve as a potential alternative therapy for stem cell transplantation.

### 3.2 CBSCs and CBSC-EV restrict cell deaths and promote angiogenesis in the myocardium post-MI

Over the years, several key hypotheses have surfaced regarding the mechanisms for cell therapy mediated augmentation of cardiac function [8,30,31]. Potential mechanisms include but are not limited to the salvage of cardiomyocytes at risk, neovascular formation and immunomodulation [[32], [33], [34], [35], [36]]. Consistent with our previous study in a large animal model [36], our present data showed that CBSCs and CBSC-EV transplantation largely reduced TUNEL positive cells in the myocardium 2 days post-MI compared with saline (Fig. 2). CBSC-EV also reduced apoptosis of NRVMs after exposure to H_2_O_2_ *in vitro* (Supplementary Fig. 1). Concurrently, CBSC-EV significantly improved tube formation of HUVECs *in vitro* (Supplementary Fig. 1) and increased the number of vWF and SM22 positive cells in the myocardium 6 weeks post-MI (Supplementary Fig. 4). Collectively, our data suggest that CBSC-EV possess the capacity to salvage myocytes at the site of injury that are challenged with acute damage and can promote vessel formation in the myocardium. This protective effect on cardiomyocytes and vessels may largely limit the expansion of infarct size and restore the blood supply of the injured area, thus improving post-MI cardiac function [37,38].

### 3.3 CBSCs and CBSC-EV induce pro-reparative immune cell polarization post-MI

The immunomodulatory abilities of cell therapy have been under intense investigation in recent decades [33,36,[39], [40], [41]]. However, whether and how extracellular vesicles from parental cells induce the same salutary effects in immune modulation after ischemic injury remains unclear. Moreover, our previous study suggested that CBSCs possess preferable reparative effect compared with other cell therapies [16]. Thus, the functional effect of CBSC-EV on immunomodulation and its unique molecular cargo are the focus of this study.

Acutely after infarction, factors from the necrosing myocardium first evoke the recruitment of neutrophils and pro-inflammatory M1 macrophages (MΦs), which facilitate the clearance of necrosed cells at the injured site [22,23,42]. After this ‘acute inflammatory phase’ (0–4 days post-MI), anti-inflammatory M2 MΦs and T-regulatory cells (Tregs) infiltrate into the injured site and promote a pro-reparative phase (4–14 days post-MI) that results in scar formation and angiogenesis [23,38]. Further tissue damage can be a consequent of prolonged or intense inflammation [42]. Thus, resolution of inflammation and progression of tissue repair can be a vital therapeutic target. In this study, we first showed that both CBSCs and CBSC-EV transplantation modulated the level of systemic inflammatory cytokines and chemokines 24 h post-MI, with reduced levels of pro-inflammatory chemokines including CXCL10, CXCL1, CCL2, CCL12, and CXCL9 in the plasma (Fig. 4). Alterations of plasma cytokine levels may be a subsequence of the immune response at the injured site [43]. Cytokines in systemic circulation mediate the recruitment of immune cells such as neutrophils and leukocytes [24]. Previous studies suggest that increased concentration of pro-inflammatory cytokines in the blood may induce inflammatory instability and adverse events after ischemic injury [44,45]. Other than the change of cytokine levels in circulation, our data also showed that CBSCs and CBSC-EV treatment altered the gene expression levels of cytokines at the injured site with decreased expression of pro-inflammatory cytokines and increased expression of an anti-inflammatory cytokines (Fig. 2), which might explain the attenuated apoptosis observed 2-day post-MI. Together, these results suggest an alteration of cytokine levels both in the microenvironment in the infarct zone and systemic circulation acutely after MI, which may contribute to the different immune profile post-injury. Our data suggested CBSCs and CBSC-EV significantly reduced the number of pro-inflammatory CD86^+^ M1 MΦs and increased the number of pro-reparative CD206+ M2 MΦs in the myocardium 5 days post-MI as determined by both histology and Flow cytometry analysis. CBSCs and CBSC-EV also exhibited the ability to modulate T-lymphocyte populations *in vivo*, with less CD8^+^ cytotoxic T cell populations (%) and increased CD4^+^ T-helper cell populations (%), specifically Tregs (Foxp3+) from 7 to 14 days post-MI (Fig. 3, Supplementary Fig. 4). The T cell modulation may also be a result of enrichment in non-coding RNAs that are capable of T cell surface receptor binding, suggested by gene set enrichment analysis on molecular function of DEGs (Fig. 5C). These findings suggest that CBSCs and CBSC-EV modulate immune cell population in the injured site and expand the immune cell populations (M2 MФs and Tregs) that contribute to the establishment of the scar and maintenance of a pro-reparative state [[46], [47], [48]]. The expedited transition from acute inflammatory phase to the pro-reparative phase may reduce infarct expansion and promote scar maturation.

### 3.4 CBSC-EVs are enriched miR-182/183 which mediate the altered macrophage phenotype

The capacity of CBSC-EV in modulating macrophage polarization and function was validated *in vitro*. CBSC-EV altered cytokine gene expression levels, inhibited M1 polarization and promoted phagocytosis in BMDMs under LPS stress (Fig. 4). To investigate the potential mechanisms, RNA-sequencing was performed to analyze the transcriptome profile in CBSC-EV. EPCs and CDCs are the most common cell therapies that are applied to ischemic heart injury. Transcriptome profile of EVs derived from EPCs and CDCs were thus analyzed. CBSC-EV showed largely distinct transcriptome profile compared with EPC and CDC derived EVs (Fig. 5). More importantly, the DEGs in CBSC-EV were significantly enriched in GO terms related with immune cell receptor binding, which may underlie the prominent immune cell response we observed both *in vivo* and *in vitro*. In particular, miR-182 and miR-183, which are considered as a cluster together with miR-96 [49], ranked at the top of the most significantly upregulated DEGs in CBSC-EV (Fig. 5E). Our *in vitro* experiment determined that miR-182/183 mimics treatment largely recapitulates the effect of CBSC-EV on BMDMs, if not all, by attenuating the gene expression levels of IL6 and CD86, and reducing the M1 macrophage population after LPS treatment (Fig. 6A–C). The immune modulation effect of CBSC-EV could be attenuated by blocking miR-182/183 (Fig. 6D). There were several studies investigating the role of miR-182 on T cells polarization [[50], [51], [52]], suggesting that miR-182 is vital for Treg differentiation. However, the role of miR-182/183 on macrophages remains unknown. Our data showed that miR-182/183 treatment promotes BMDMs metabolic reprograming to an anti-inflammatory phenotype, with signatures of preserved oxygen consumption and reduced ROS level [53,54]. To understand how miR-182/183 promotes anti-inflammatory metabolic reprograming, we first determined Rasa1 as a target of miR-182-5p by Western blot and luciferase assay (Fig. 7A). Then we showed that miR-182/183 treatment promotes Ras downstream ERK/c-Myc pathway by inhibiting Rasa1 (Fig. 7C). ERK is essential in preserving oxygen consumption in macrophages, inhibition of ERK will lead to reduced oxygen consumption [55]. Thus, miR-182/183 treatment may preserve OCR with the preserved activation of ERK. c-Myc expression is restricted to M2 phenotype [56], a switching from c-Myc to HIF1α pathway leads to pro-inflammatory polarization [57]. Moreover, previous paper suggested that c-Myc targets SOD2 promoter and controls SOD2 expression [58], which is a major enzyme that handles ROS in the mitochondrial matrix. The activation of c-Myc and SOD2 in miR-182/183 treated BMDMs may explain the reduction of mitochondrial and cellular ROS presented in Fig. 6G. Moreover, miR-182/183 treatment also preserved Akt expression by inhibiting Rasa1. Previous publication suggested that Akt activation promotes the anti-inflammatory polarization of macrophages under LPS stimuli [59,60]. Taken together, miR-182/183, by inhibiting Rasa1, activates Ras/ERK/c-Myc and Ras/Akt pathway, that increases oxygen consumption and reduces ROS levels to promote an anti-inflammatory phenotype in response to LPS stress (Fig. 7D).

## 4 Conclusion

**Fig. 8:**
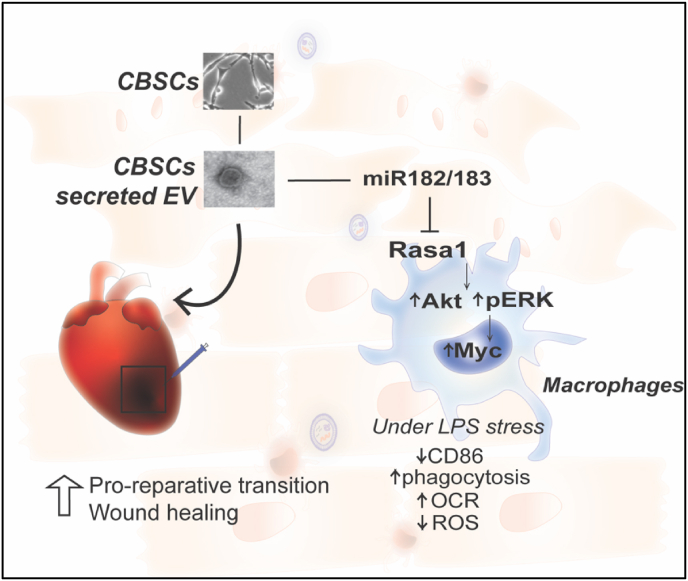
Illustration of the reparative mechanisms of CBSC-EV.

Our study showed that CBSCs derived extracellular vesicles possess equivalent reparative effect as CBSCs in augmenting cardiac function and restricting scar formation after MI in a mouse model. The potential mechanism of enhanced cardiac repair with CBSC-EV treatment is through the following mechanisms: 1) CBSC-EV contains unique transcript cargoes that are enriched in non-coding RNAs, including but not limited to miR-182/183, which may bind to immune cell surface receptors and promotes anti-inflammatory polarization. Thus, shortening the pro-inflammatory phase and shifting the immune cell landscape to the pro-reparative state after ICI. 2) CBSC-EV spares viable cardiomyocytes at border zone and attenuates cell apoptosis, restricting infarct expansion thus promotes cardiac repair (Fig. 8).

## 5 Methods

### 5.1 Statistics

All data were expressed as a mean ± SD. Two-sided testing was used for all statistical tests. For the *in vitro* tube formation assay, comparisons for data with single measurement within two groups were performed using unpaired *t*-test. Comparison between multiple groups were done by 1-way ANOVA, followed by Tukey's post-hoc multiple comparisons test. For echocardiography parameters with repeated measures over time, analyses were performed by 2-way ANOVA, followed by Tukey's multiple comparison test, to test treatment group differences at each time point as well as change vs. baseline over time within each treatment group. P value ≤ 0.05 was considered as statistically significant. Statistical analysis was performed using GraphPad prism software (Version 7.0 d, GraphPad Inc., La Jolla, CA).

## Study approval

All animal procedures were approved by the Temple University Lewis Katz School of Medicine Institutional Animal Care and Use Committee.

## Data availability

The data, methods used in the analysis, and materials used to conduct the research will be made available to any researcher for purposes of reproducing the results.

## Author contributions

Y.Y., S.M. and S.R.H. conceived and designed the research studies. S.M., Y.Y., J.J., C.D.T., E.A.F., L.K, E.M., T.K., D.E., T.W., M.W., L.M., C.B., M.W., H.K. conducted experiments and acquired data, S.M., Y.Y. analyzed the data. Y.Y., J.J. drafted the manuscript with the input from S.M., S.R.H., M.K., R.K. S.M. and S.R.H. made critical revision of the manuscript for key intellectual content. All authors discussed the results and commented on the manuscript.

## Declaration of competing interest

The authors declare that S. R. Houser is a named inventor on intellectual property filings that are related to the cortical bone stem cells used in this study. In addition, S. R. Houser is a cofounder and scientific advisor and holds equity in MyocardTherapeutics, LLC, a biotech startup which will license S. R. Houser’s cortical bone cell technology from Temple University for commercial development and clinical trials. MyocardTherapeutics, LLC, has not funded any aspect of this research. S. Mohsin is a named inventor on intellectual property filings that are related to the cortical bone stem cells used in this study.
