# Supplementary material for: miR-182/183-Rasa1 axis induced macrophage polarization and redox regulation promotes repair after ischemic cardiac injury
Source: Redox Biol. 2023 Sep 29;67:102909. doi: 10.1016/j.redox.2023.102909 (PMC10570148; doi:10.1016/j.redox.2023.102909)
Supplement: Multimedia component 1 [file mmc1.docx]

**Supplementary Materials**

**Methods**

**Supplementary Tables and Figures**

Supplementary Figure 1. Extracellular vesicles (EVs) isolated from CBSCs

Supplementary Figure 2. Cardiac function analysis by echocardiography before and 6 weeks post-MI.

Supplementary Figure 3. Gating strategy for the analysis of T cell population.

Supplementary Figure 4. CBSCs and CBSC-EV induced T cell polarization post-MI.

Supplementary Figure 5. CBSCs and CBSCs derived extracellular vesicles (CBSC-EV) reduced apoptosis and promoted angiogenesis in the myocardium after MI.

Supplementary Figure 6. Gating strategy for the analysis of macrophage population.

Supplementary Figure 7. Heatmap of the most significantly different genes in EVs isolated from CBSCs, CDC and EPC.

Supplementary Figure 8. miR182/183 attenuated cellular ROS production in BMDMs after 3h LPS treatment.

Supplementary Figure 9. CBSC-EV promote T cell polarization *in-vitro*.

Supplementary Table I. Primer List

Supplementary Table II. Antibody List

**Methods**

**Cortical Bone Stem Cell (CBSCs) isolation**

Cortical bone stem cells were isolated from tibias and femurs of eGFP+ C57BL/6 mice as described previously^1^. Briefly, the epiphyses of the bones were removed, and the bone marrow was flushed out by three times washing with phosphate-buffered saline (PBS). The remaining cortical bone was crushed using a sterilized mortar and pestle, bone fragments (around 3mm) was further digested in collagenase II for an hour and passed through 100μm and 40μm filters. The remaining cells were plated in CBSCs growth media: DMEM/F12 Media (Lonza/Biowhittaker; Basel, Switzerland) + 10% fetal bovine serum (Gibco Life Technologies; Grand Island, NY), 1% Penicillin/Streptomycin/L-glutamine (Gibco Life Technologies; Grand Island, NY), 0.2% insulin-transferrin-selenium (Lonza; Basel, Switzerland), 0.02% basic-fibroblast growth factor (Peprotech; Rock Hill, NJ), 0.02% epidermal growth factor (Sigma; St. Louis, MO), and 0.01% leukemia inhibitory factor (Millipore; Billerica, MA) until homogenous population of stem cells was obtained as described previously^2^. The CBSCs homogenous population were confirmed by their unique morphology as showed in Supplementary Figure 1A, which are homogenous spindle-shaped cells. CBSCs have distinct identity and largely different from other stem cell types. The characteristics of CBSCs including cell size, cell shape, proliferation capacity, cell surface marker, etc., were characterized and depicted in previous publication^1^.

**Extracellular Vesicles Isolation and Characterization:**

Extracellular vesicles (EVs) were isolated and characterized as described previously^3, 4^. CBSCs isolated from C57BL/6 mice (passage 10) were cultured in 10 cm2 dishes at a density of 150,000 cells/dish in exosome-depleted FBS media (System Bio, Exo-FBS-250A-1). After 3 days in culture, the supernatant containing secreted extracellular vesicles was collected and filtered through a 0.22-µM media sterilizer filter (Corning, 430517). The filtered supernatant was concentrated in 100,000 media concentrator conicals via centrifugation at >1,000 g for 30 min. After centrifugation, the concentrate was collected and pipetted into 25 mL glass ultracentrifugation conicals. To remove any cellular debris, the concentrate was ultracentrifuged at 20,000 g for 30 min. Following the removal of cellular debris, the media was collected and slowly layered on top of 4 mL of sucrose gradient (pH = 7.4). The media + sucrose gradient was ultracentrifuged at 100,000 g for 1 h to pull the exosomes into the sucrose gradient. Following the ultracentrifugation, the media above the exosome layer was removed and 1× PBS was added to the conical at the equal volume of the previously removed media. The remaining solution within the conicals was then mixed to dissipate the gradient effect of the remaining sucrose. The conicals were then ultracentrifuged again at 100,000 *g* for 1 h to precipitate out the exosomes into a pellet. The exosome protein concentration was measured via Bradford Concentration Assay (BCA), and the exosome particle size and concentration were measured via Nanosight assay.

EVs from endothelial progenitor cells (EPCs) and cardiosphere derived cells (CDCs) were isolated as previously described^5^. EPCs were isolated from 8–10-week-old C57BL/6J mouse BM from tibiae, femurs, and hip bones and cultured in endothelial cell basal medium-2 (EBM-2, Lonza) supplemented with growth factors (EGM-2 SingleQuots, Lonza) and 10% exosome-free FBS. Media were collected every day from day 4 to day 10. CDCs were isolated from mouse heart based on c-kit expression described previously^6^ and cultured in DMEM/F12 and neurobasal medium (1:1) supplemented with 10% exosome-free FBS, L-glutamine (2mM), insulin-transferrin-selenium (ITS) (1%), penicillin-streptomycin (P/S), B27 (1%), N2 (1%), and growth factors (bFGF, EGF, and LIF). EVs were collected from exosome-free FBS media of EPCs and CDCs, and isolated by ultracentrifugation method as described above.

**Preparation and Treatment of NRVMs**

Neonatal rat ventricular myocytes (NRVMs) were isolated from 1-2 days old rat pups as previously described^7^ and cultured in F-10 medium (Gibco) supplemented with 10% Fetal Bovine Serum (FBS). For Apoptotic Stimuli, 50µM of H_2_O_2_ was used as described earlier^8^. Samples were then immunostained to quantify the number of apoptotic NRVMs using DeadEnd Fluorometric TUNEL system (Promega Madison, WI) according to the manufacturer’s protocol.

**Matrigel Tube Forming Assay**

HUVECs were seeded in a 48-well plate coated with growth factor-reduced Matrigel (BD Biosciences, CA, USA) and treated with extracellular vesicles overnight. Tube formation was examined by phase- contrast microscopy 24 hours later. The Tube formation was quantified using Image J.

**Real-Time Reverse Transcriptase-Polymerase Chain Reaction**

Total RNA was isolated from cells and heart tissues 1-day post-MI using Quick-RNA MiniPrep (Zymo Research, CA) according to manufacturer’s protocol. cDNA was prepared using iScript cDNA Synthesis Kit (Bio Rad, CA). Real-time polymerase chain reaction (PCR) was performed on samples in triplicate using iQ SYBER Green (Qiagen, CA). Primer sequences are listed in Supplementary Table I.

**Induction of Acute MI, Delivery of CBSCs/ CBSC-EVs and Echocardiography**

All surgical procedures and animal care protocols were approved by the Temple University Institutional Animal Care and Use Committee. Animals (C57BL/6 8-12 weeks old male mice) were divided into three groups (n=25/group) receiving either Saline, CBSCs or Extracellular vesicles derived from CBSCs (CBSC-Exo). Animals underwent myocardial infarction procedure by permanent ligation of the left anterior descending artery (LAD) as described previously^2^. Once the LAD was ligated, the LAD-perfused area, which is the lower left part of the left ventricle, turned pale. Border zone is defined as the boundary between the injured, hypoxic, pale color tissue and adjacent viable, normoxic, dark red color tissue. CBSCs (100,000 cells, 4000 cells/μL) or CBSC-Exo (7x10^8^ particles/mL) were injected intramyocardial after MI at 5 injection sites in the border zone area. The animals were sacrificed at Day 1, 2, 5, 7, and 14 for FACS analysis and histological analysis, and week 6 for functional and histological data analysis.

Mice underwent serial echocardiography at baseline, 1-, 2-, 4- and 6-week post-surgery as described previously^2, 9^. In brief, Transthoracic two-dimensional echocardiography was performed using the Vevo2100 (VisualSonics, Toronto, ON, Canada) equipped with a 30-MHz transducer to record B and M-mode measurements of both long- and short-axis of the heart in mice under anesthesia.

**Proteome Profiler Assay**

Blood was collected 24 hours post-surgery from animals treated with saline, CBSCs and CBSC-Exo. Plasma was collected as described in the manufacturer’s protocol using heparin as an anticoagulant. Samples were prepared by centrifuging for 15 minutes at approximately 2000 x g within 30 minutes of collection. Samples were then assessed using a Proteome Profiler Mouse Cytokine Array Kit (R&D systems, Catlog #: ARY006) using manufacturer’s protocol. The data was analyzed using image J.

**Immune Cell Isolation from Heart and FACS staining**

Hearts were collected for isolation of immune cells. Briefly, hearts were cut into small pieces and then incubated in a digestion buffer containing 20mM Hepes/PBS, Collagenase Type I 450U/ml, Deoxyribonuclease I 60U/ml, Hyaluronidase. 60U/ml, and Collagenase, Type XI 125U/ml. Hearts were incubated 37°C for 60 minutes then passed through a 40um cell strainer, then centrifuged for 3 minutes at 50g to remove cardiomyocytes. The supernatant was centrifuged at 500g to collect immune cells and proceeded to staining with antibodies for FACS listed in Supplementary Table II. The samples were run on FACS for CD45, CD206, CD68, CD4, CD8 and Foxp3.

**Histological Analysis**

Paraffin-embedded samples fixed at 6 weeks post-MI were stained with Masson’s Trichrome (Sigma-Aldrich; St. Louis, MO) for quantification of infarct size (n=8) in each group. Masson’s Trichrome-stained bright field micrographs were acquired using a light microscope (Olympus BX40) and the analysis was performed using ImageJ as previously described^10^. Wheat Germ Agglutinin (WGA) staining was also performed on sections fixed at 6 weeks post-MI to determine the cross-sectional area of cardiomyocytes. The analysis was performed using ImageJ. Histological sections were stained for SM22 (ab14106, ABCAM) and vWF (ab6994, ABCAM) together with α-sarcomeric actin (A2172, Sigma) to detect angiogenesis after CBSCs and CBSCs derived exosome treatment 6 weeks after transplantation. Sections were also stained for CD86 (ab119857, ABCAM), CD206 (R&D system, AF2535) and CD3 (ab5690, ABCAM) 5 and 14 days after transplantation to detect M1 macrophage and T cell influx after transplantation.

Terminal deoxynucleotidyl transferase-mediated deoxyuridine triphosphate nick-end labeling (TUNEL) was performed on paraffin-embedded tissues fixed at 48 hours after MI. Samples were then immunostained to quantify the number of apoptotic myocytes using DeadEnd Fluorometric TUNEL system (G3250, Promega Madison, WI). Immunostaining for cardiac contractile proteins was performed using mouse IgG anti-sarcomeric actin (Sigma-Aldrich).

# EdU staining was performed using Click-iT™ EdU Cell Proliferation Kit for Imaging (C10337, Life Technologies) following manufacturer’s instructions.

**Mouse Bone Marrow Derived Macrophage Isolation and Treatment**

Macrophages were derived from 2-4 months old C57BL/6 male mice using as previously described^11^. Briefly, bone marrow was flushed out from the femur and tibia. After lysing red blood cells, macrophages were plated in RPMI medium (ThermoFisher, 72400047) with 50ng/mL macrophage-colony stimulating factor (M-CSF, Pepro Tech, 315-02) for 6-7 days for macrophage differentiation in a 6-well dish. BMDMs were treated with RPMI (ThermoFisher, 72400047) or CBSC-EV for 24 hours to determine the effect of CBSC-EV at baseline level. In addition, BMDMs treated with RPMI or CBSC-EV were treated with 50ng/ml LPS (lipopolysaccharide, Invitrogen, 00-4976-03) for 18 hours to induce inflammatory response. BMDMs were either collected for RNA isolation to detect gene expression levels of Spp1, IL18, IL6, etc, or collected for flow cytometry analysis. BMDMs were also incubated with phagocytosis beads for 1 hour before collection for flow cytometry analysis. To investigate the effect of miR182/183, BMDMs were treated with LPS together with 10nmol/L miR182+miR183 mimics or negative control (Dharmacon, C-310437-06-0020, C-310436-07-0020, CN-001000-01-20) for 24 hours. The effect of miR182 and miR183 was also validated by an addition of miR182 and miR183 inhibitor (Horizon/PerkinElmer IH310436-08-0010, IH310437-07-0010) to CBSC-EV treatment. BMDMs were collected after treatment for RNA isolation, flow cytometry analysis and immunoblotting respectively. For flow cytometry analysis, BMDMs were stained with surface marker antibodies F4/80, CD86, CD80 listed in Supplementary Table II. For immunoblotting, protein samples were isolated from cell lysates. Antibodies used are GAPDH (Sigma, MAB374), Akt (Cell signaling, 2920S), phosphorylated Akt (Cell signaling, 4050S), phosphorylated ERK1/2 (Cell signaling, 9101), c-Myc (Cell signaling, 5606), SOD2 (Cell signaling, 13141), Catalase (Cell signaling, 14097).

**Spleen Derived T Cells Isolation and Treatment**

T cells were derived from spleens from 2-4 months old C57BL/6 male mice using Stel Cell Tech Isolation kit (STEMCELL Technologies, 19852A). Briefly, spleens were mashed and lysed to remove red blood cells. Cells were incubated with CD4+ isolation cocktail and then filtered with magnetic beads. CD4+ T cells were cultured in T cell culture medium supplemented with 30U/ml IL-2 in a 96 well v-bottom plate for 24 hours before treated with miRNA mimics and negative control. T cells were collected for flow cytometry staining 24 hours after treatment. T cell surface markers were identified with antibodies CD45, CD3, CD4, CD8, CD25 and FoxP3 at the concentration listed in Supplementary Table II. Flow cytometry analysis of T cell population was acquired with LSRII Flow Cytometer (BD). Population dynamic percentages were quantified using Flow Jo software.

**RNA sequencing and Bioinformatics Analysis**

The isolation, purification of total RNA from EVs from CBSCs, EPCs and CPCs were performed by System Biosciences (SBI).  The NextSeq High Output single-end sequencing ran at SR75 using NextSeq 500/550 High Output v2 kit (Cat #FC-404-2005, Illumina, San Diego, CA, USA) was performed according to the manufacturer’s instructions. The sequence reads were analyzed and mapped as previously described^5^. The differential expression between samples was calculated by R Bioconductor package DESeq2. Genes with false discovery rate (FDR) <0.05 and fold-change (FC) ≥ 2 were considered as differentially expressed genes (DEGs). Volcano plots were generated with R Bioconductor package EnhancedVolcano. Gene Ontology gene set enrichment analysis was performed using R Bioconductor clusterProfiler package. Predicted target of miRNAs were identified through TargetScan (<https://www.targetscan.org/cgi-bin/targetscan/vert_80/view_gene.cgi?rs=ENST00000355622.6&taxid=10090&showcnc=0&shownc=0&shownc_nc=&showncf1=&showncf2=&subset=1>).

**Mitochondrial and cellular ROS assay**

BMDMs were isolated and plated in 96-well dishes for the ROS detection assay. After 7-day pre-culturing with M-CSF, BMDMs were pre-treated with miR182/183 or miRNA negative control for 24h with lipofectamine, then stimulated with LPS. Mitochondrial ROS and cellular ROS were detected following manufacture’s protocol (Abcam, ab219943, ab186027).

**Luciferase reporter assay**

HEK293 cells were transfected with mouse Rasa1 3’-UTR and control luciferase reporter plasmids (GeneCopoeia, MmiT038403-MT06, CmiT000001-MT06) followed by treatment with miR182/183 mimics for 72 hours. After 72 hours, Firefly and Renilla luciferase reporter activities were measured using Luc-Pair Duo-Luciferase Assay kit (GeneCopoeia, LF001) according to the manufacturer’s instructions.

**
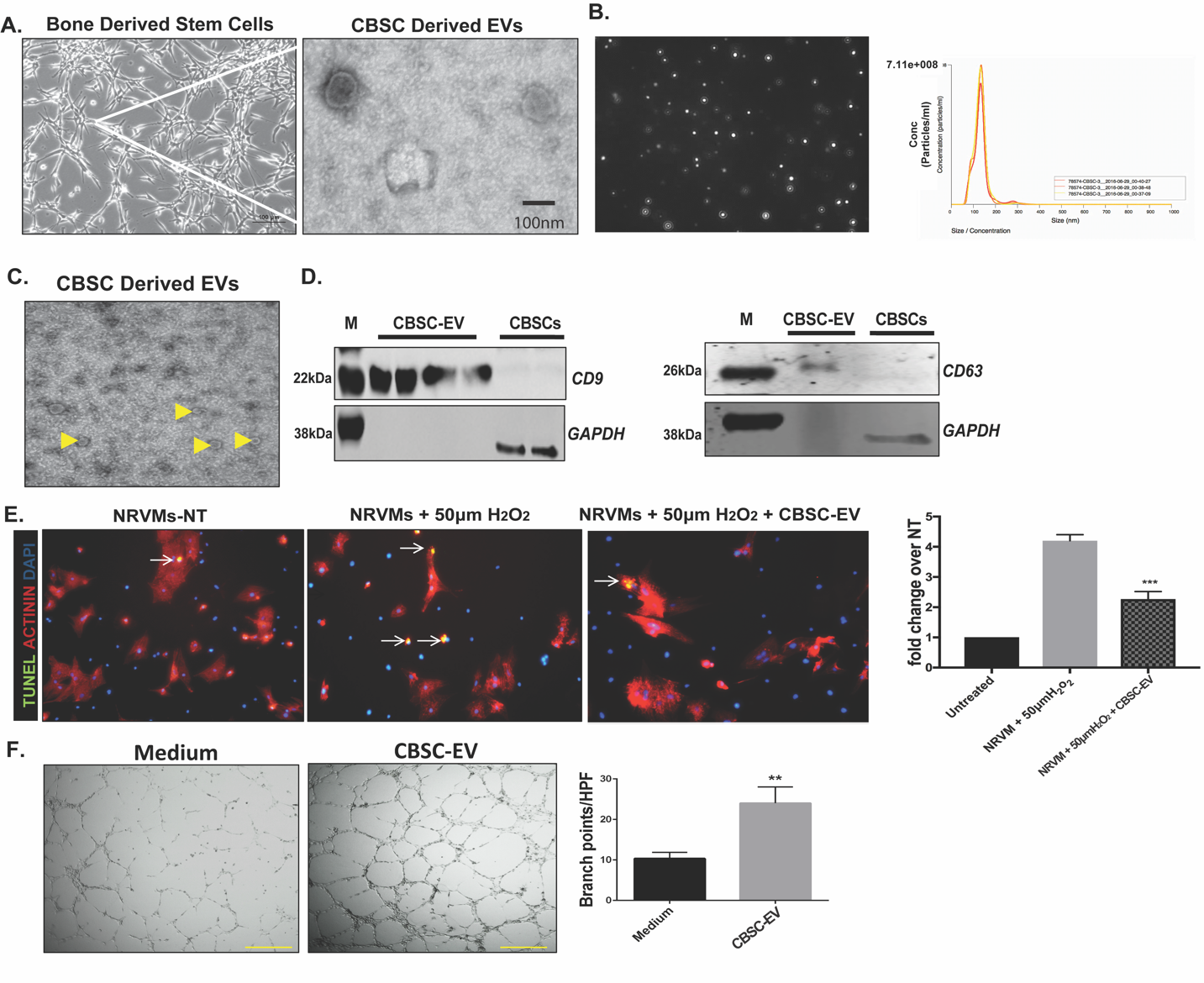
**

**Supplementary Figure 1: Extracellular vesicles (EVs) isolated from CBSCs.**

A. Bone derived stem cells and their extracellular vesicles in culture, B. Nanosight confirming the size range and particle number of CBSCs derived EVs preparation. C. Electron micrograph of CBSC derived EVs demonstrating typical morphology and size <100nm, with D. protein expression of typical exosome markers CD9 and CD63. E. TUNEL staining in NRVMS of NRVMS non treated (NT), NRVMS exposed to apoptotic injury, and NRVMS treated with CBSCs derived extracellular vesicles (CBSC-EV) and exposed to apoptotic challenge, *** *P*<0.001 CBSC-EV+50um H_2_O_2_ vs. 50um H_2_O_2_. F. Enhanced tube formation in human umbilical vein endothelial cells (HUVECs) treated with CBSC-EV versus Control media, ***P*<0.01.


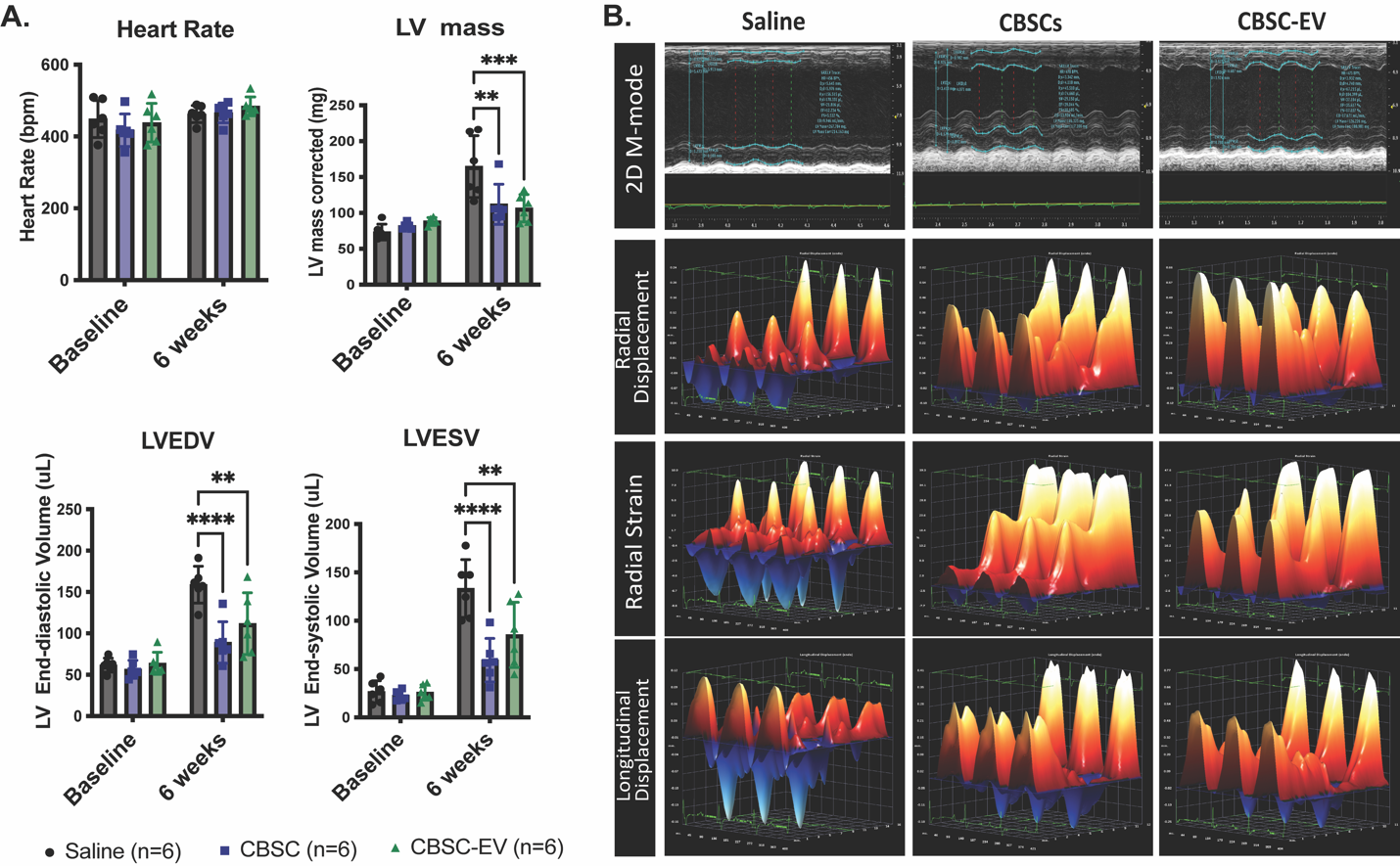


**Supplementary Figure 2: Cardiac function analysis by echocardiography before and 6 weeks post-MI.**

A. Echocardiography derived parameters: heart rate, left ventricular internal diameter (LVID), left ventricular end-diastolic volume (LVEDV), left ventricular end-systolic volume (LVESV) and left ventricular (LV) mass at baseline and 6 weeks post-MI in saline, CBSCs and CBSCs derived extracellular vesicles (CBSC-EV) treated animals. (n=6 animals per group). ***P*<0.01, ****P*<0.001, *****P*<0.0001 versus saline. B. Representative images of 2D M-mode, 3D regional wall strain diagrams in saline, CBSCs and CBSC-EV treated animals at terminal.


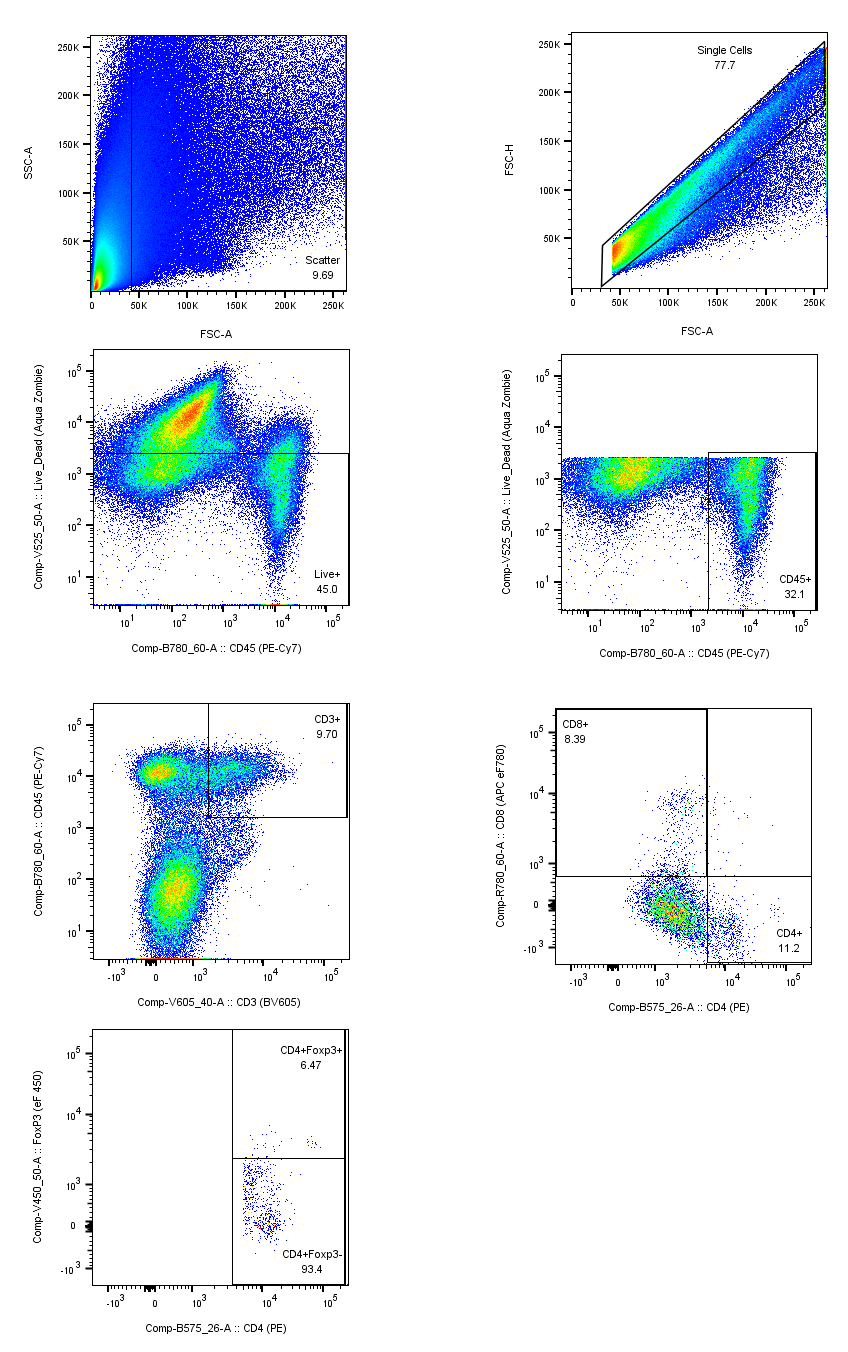


**Supplementary Figure 3: Gating strategy for the analysis of T cell population.**

Representative flow cytometry gating for T cell population from isolated cardiac cells.


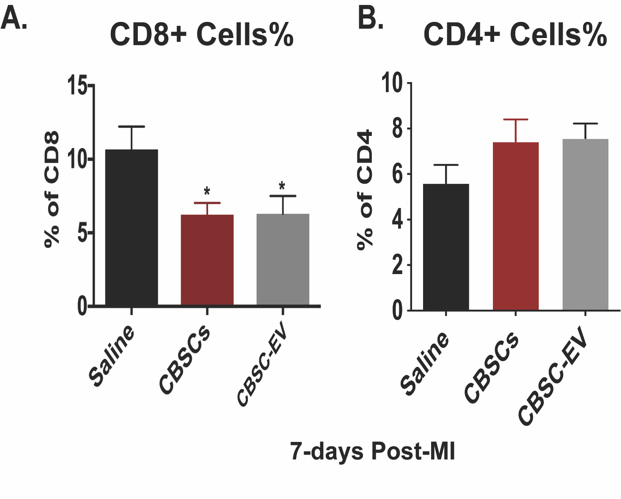


**Supplementary Figure 4: CBSCs and CBSC-EV induced T cell polarization post-MI.**

Percentage of A. CD8 and B. CD4 positive cells in total CD45 positive cell population 7 days post-MI determined by FACS. **P*<0.05, versus saline.


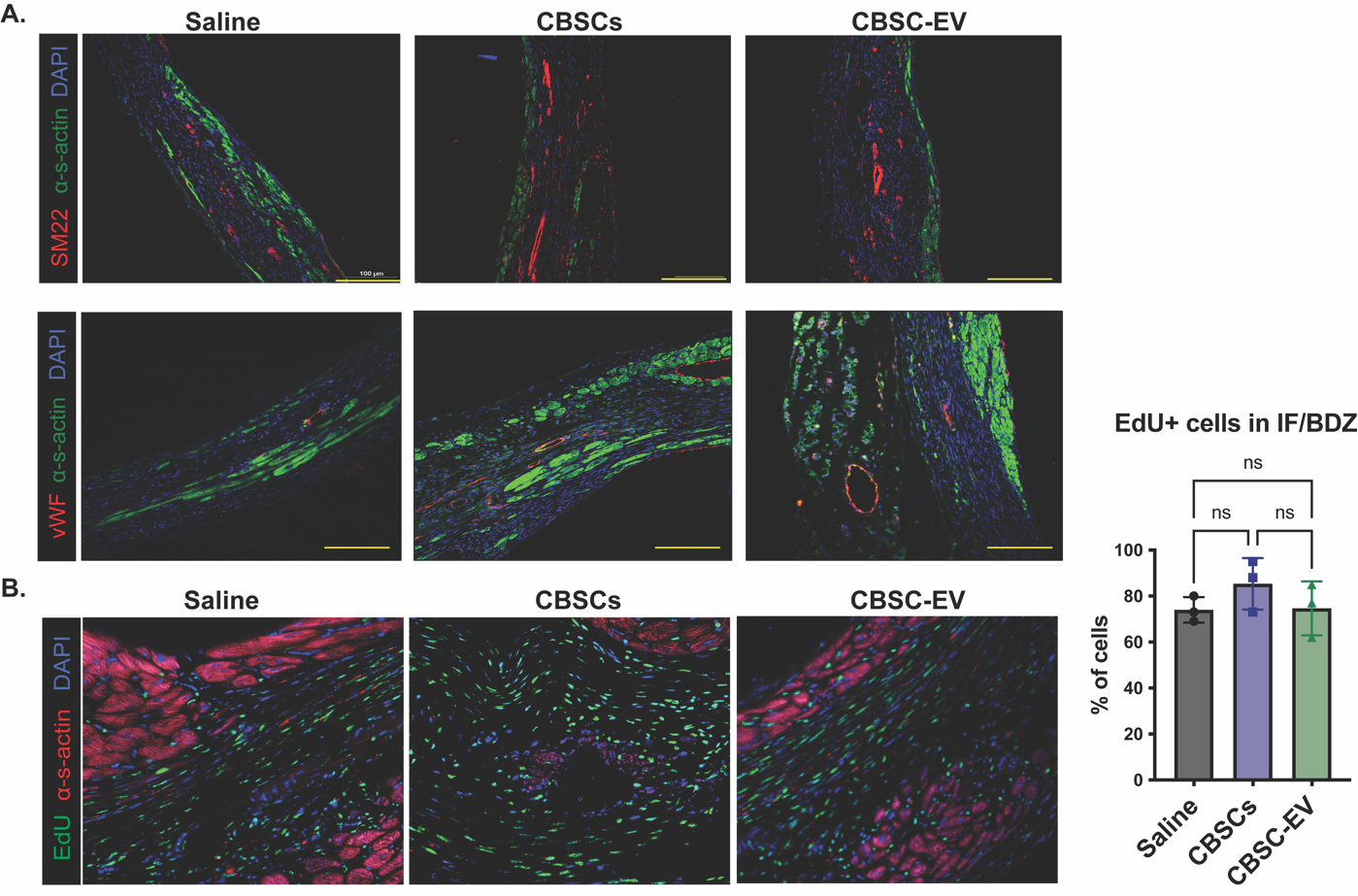


**Supplementary Figure 5: CBSCs and CBSCs derived extracellular vesicles (CBSC-EV) reduced apoptosis and promoted angiogenesis in the myocardium after MI.**

A. Immunofluorescent staining for SM22 and von Willebrand factor (vWF) (red), α-sarcomeric actin (green), and DAPI (blue). Scale bar is 100um. B. Histological analysis of 5-ethynyl-2´-deoxyuridine (EdU) positive cells at infarct/border zone after transplantation of CBSCs and CBSC-EV 6 weeks post-MI.

**
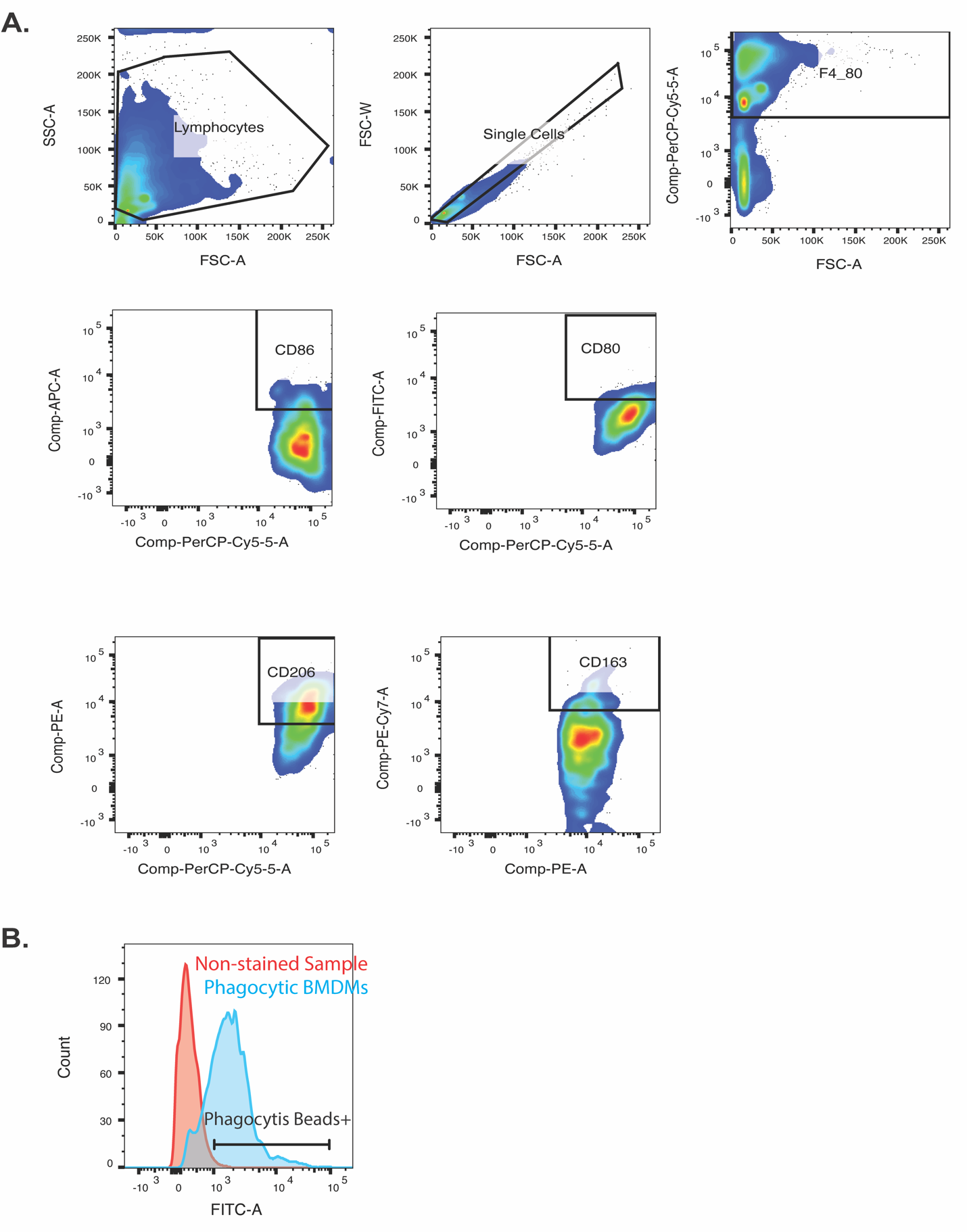
**

**Supplementary Figure 6: Gating strategy for the analysis of macrophage population.**

A. Representative flow cytometry gating for non-treated (NT) bone marrow derived macrophages. B. Gating strategy for phagocytic BMDMs treated with phagocytotic beads.


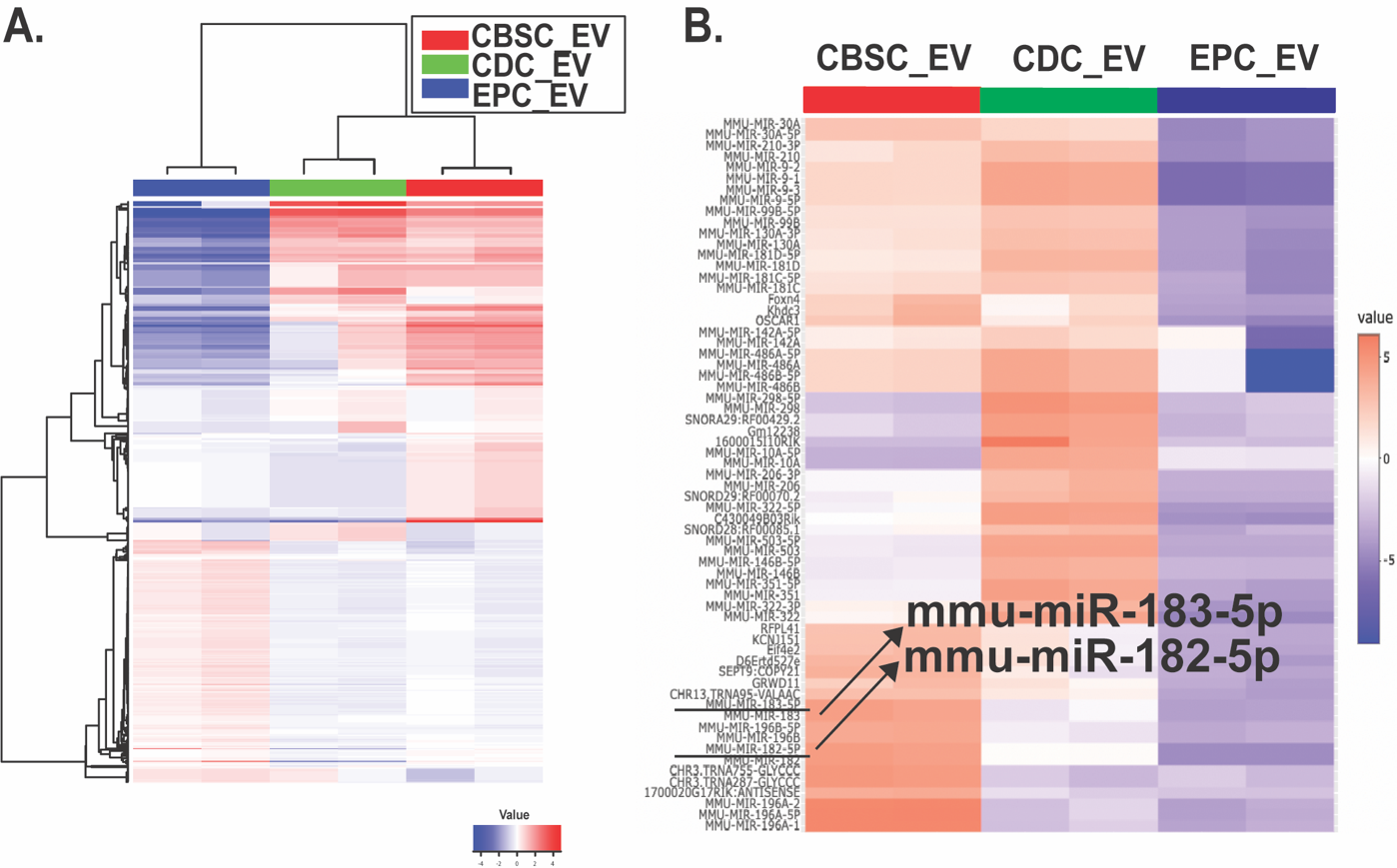


**Supplementary Figure 7: Heatmap of the most significantly different genes in EVs isolated from CBSCs, CDC and EPC.**

A: Heatmap of top 480 most differentially expressed genes. B. Heatmap of top 65 most variable genes with gene symbols.


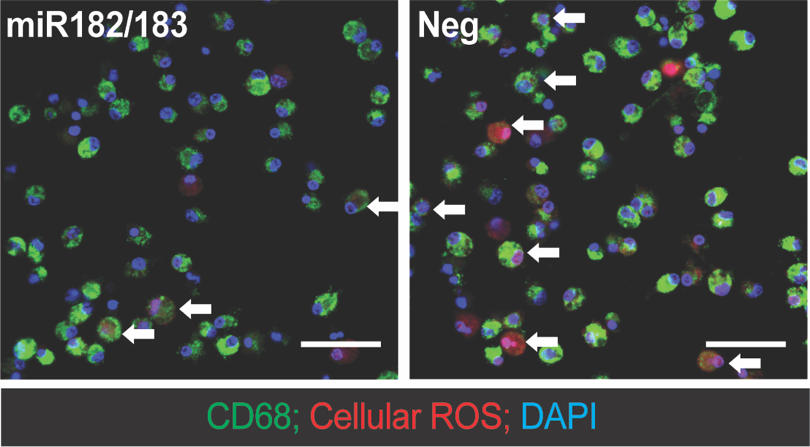


**Supplementary Figure 8: miR182/183 attenuated cellular ROS production in BMDMs after 3h LPS treatment.**

BMDMs were pre-treated with miR182/183 or miRNA negative control for 24h, then stimulated with 3h LPS treatment. Cellular ROS were detected with red dye ROS sensor.

**
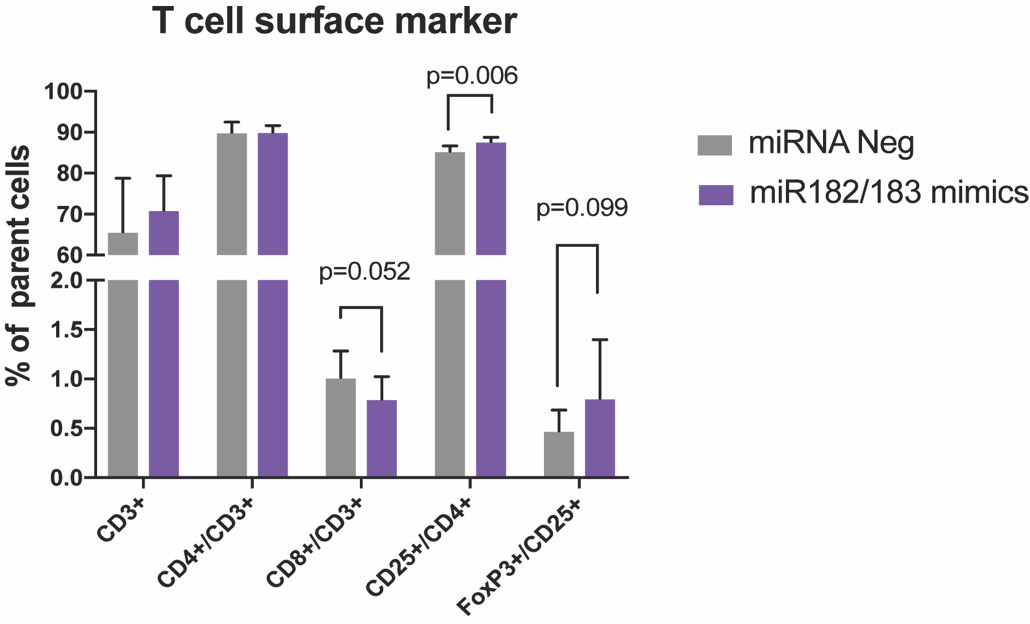
**

**Supplementary Figure 9: CBSC-EV promote T cell polarization *in-vitro*.**

A. T cells were isolated from spleen and treated with miR182/183 mimics or miRNA negative control in culture. T cell populations were determined with flow cytometry analysis.

Supplementary Table I. Primer List

|  | Forward | Reverse |
| --- | --- | --- |
| 18S | 5’-CGAGCCGCCTGGATACC | 5’-CATGGCCTCAGTTCCGAAAA |
| IL-18 | 5’-AAGAAAGCCGCCTCAAACCT | 5’-CATTGTTCCTGGGCCAAGAGG |
| IL-13 | 5’-GCTTATTGAGGAGCTGAGCAACA | 5’-GGCCAGGTCCACACTCCATA |
| IL-4 | 5’- ATGGATGTGCCAAACGTCCT | 5’-AAGCACCTTGGAAGCCCTAC |
| IL-6 | 5’-GACAAAGCCAGAGTCCTTCAGA | 5’-TGTGACTCCAGCTTATCTCTTGG |
| IL-10 | 5’-GGTTGCCAAGCCTTATCGGA | 5’-GGGGCATCACTTCTACCAGG |
| SPP1 | 5’-CCTTGCTTGGGTTTGCAGTC | 5’-CAGGCTTACCTTGGCTGGTT |
| CD86 | 5’-AAGCAGACGCGTAAGAGTGG | 5’-TGGTTGTTCAAGTCCGTGCT |

Supplementary Table II. Antibody List

| Antibody | Cat# | Company | Antibody Dilution (/100ul) |
| --- | --- | --- | --- |
| Aqua Zombie | 423101 | BioLegend | 0.13ul |
| CD45 PE/CY7 | 25-0451-82 | Invitrogen | 1.25ul |
| CD3 BV605 | 100237 | BioLegend | 1.25ul |
| CD4 PE | 12-0041-82 | Invitrogen | 1.25ul |
| CD8 APC eflour789 | 47-0041-82 | Invitrogen | 1.25ul |
| CD25 AF647 | 563598 | BD Pharmigen | 1.25ul |
| FoxP3 ef450 | 48-5773-82 | BioLegend | 0.5ul |
| F4/80 PerCP-Cy5.5 | 45-4801-82 | Invitrogen | 1ul |
| CD80 FITC | 11-0801-85 | Invitrogen | 1ul |
| CD86 APC | 558703 | BD Pharmingen | 1ul |
| CD206 PE | 12-2061-82 | Invitrogen | 1ul |
| CD163 PE-Cyanine7 | 25-1631-82 | Invitrogen | 1ul |
| Phagocytosis Assay kit | 600450 | Cayman |  |

**References**

1. Mohsin S, Troupes CD, Starosta T, Sharp TE, Agra EJ, Smith S, Duran JM, Zalavadia N, Zhou Y, Kubo H, Berretta RM and Houser SR. Unique Features of Cortical Bone Stem Cells Associated With Repair of the Injured Heart. *Circ Res*. 2015;117:1024-33.

2. Duran JM, Makarewich CA, Sharp TE, Starosta T, Zhu F, Hoffman NE, Chiba Y, Madesh M, Berretta RM, Kubo H and Houser SR. Bone-derived stem cells repair the heart after myocardial infarction through transdifferentiation and paracrine signaling mechanisms. *Circ Res*. 2013;113:539-52.

3. Greening DW, Xu R, Ji H, Tauro BJ and Simpson RJ. A protocol for exosome isolation and characterization: evaluation of ultracentrifugation, density-gradient separation, and immunoaffinity capture methods. *Methods Mol Biol*. 2015;1295:179-209.

4. Khan M, Nickoloff E, Abramova T, Johnson J, Verma SK, Krishnamurthy P, Mackie AR, Vaughan E, Garikipati VN, Benedict C, Ramirez V, Lambers E, Ito A, Gao E, Misener S, Luongo T, Elrod J, Qin G, Houser SR, Koch WJ and Kishore R. Embryonic stem cell-derived exosomes promote endogenous repair mechanisms and enhance cardiac function following myocardial infarction. *Circ Res*. 2015;117:52-64.

5. Huang G, Garikipati VNS, Zhou Y, Benedict C, Houser SR, Koch WJ and Kishore R. Identification and Comparison of Hyperglycemia-Induced Extracellular Vesicle Transcriptome in Different Mouse Stem Cells. *Cells*. 2020;9.

6. Fransioli J, Bailey B, Gude NA, Cottage CT, Muraski JA, Emmanuel G, Wu W, Alvarez R, Rubio M, Ottolenghi S, Schaefer E and Sussman MA. Evolution of the c-kit-positive cell response to pathological challenge in the myocardium. *Stem Cells*. 2008;26:1315-24.

7. Hullmann JE, Grisanti LA, Makarewich CA, Gao E, Gold JI, Chuprun JK, Tilley DG, Houser SR and Koch WJ. GRK5-mediated exacerbation of pathological cardiac hypertrophy involves facilitation of nuclear NFAT activity. *Circ Res*. 2014;115:976-85.

8. McLaughlin S, McNeill B, Podrebarac J, Hosoyama K, Sedlakova V, Cron G, Smyth D, Seymour R, Goel K, Liang W, Rayner KJ, Ruel M, Suuronen EJ and Alarcon EI. Injectable human recombinant collagen matrices limit adverse remodeling and improve cardiac function after myocardial infarction. *Nat Commun*. 2019;10:4866.

9. Yang Y, Schena GJ, Wang T and Houser SR. Postsurgery echocardiography can predict the amount of ischemia-reperfusion injury and the resultant scar size. *Am J Physiol Heart Circ Physiol*. 2021;320:H690-H698.

10. Kraus L, Ma L, Yang Y, Nguyen F, Hoy RC, Okuno T, Khan M and Mohsin S. Cortical Bone Derived Stem Cells Modulate Cardiac Fibroblast Response via miR-18a in the Heart After Injury. *Front Cell Dev Biol*. 2020;8:494.

11. Hobby ARH, Berretta RM, Eaton DM, Kubo H, Feldsott E, Yang Y, Headrick AL, Koch KA, Rubino M, Kurian J, Khan M, Tan Y, Mohsin S, Gallucci S, McKinsey TA and Houser SR. Cortical bone stem cells modify cardiac inflammation after myocardial infarction by inducing a novel macrophage phenotype. *Am J Physiol Heart Circ Physiol*. 2021;321:H684-H701.
